# Plants used for the management of venereal diseases in sub-Saharan Africa: a systematic review and critical assessment of their research status

**DOI:** 10.1186/s41182-024-00651-y

**Published:** 2024-12-26

**Authors:** Temitope O. Omogbene, Ibraheem O. Lawal, Stephen O. Amoo, Anne A. Adam, Fikisiwe C. Gebashe, Adeyemi O. Aremu

**Affiliations:** 1 School of Life Sciences, College of Agriculture, Engineering and Science, University of KwaZulu-Natal, Westville, 4001 South Africa; 2 Biomedicinal Research Centre, Forestry Research Institute of Nigeria, Jericho Hill, P.M.B 5054, Ibadan, 200272 Nigeria; 3 Agricultural Research Council – Vegetables, Industrial and Medicinal Plants, Private Bag X293, Roodeplaat, Pretoria, 0001 South Africa; 4 Unit for Environmental Sciences and Management, Faculty of Natural and Agricultural Sciences, North-West University, Private Bag X6001, Potchefstroom, 2520 South Africa; 5 Indigenous Knowledge Systems Centre, Faculty of Natural and Agricultural Sciences, North-West University, Private Bag X2046, Mmabatho, 2790 South Africa

**Keywords:** Antimicrobial, Biodiversity, Ethnobotany, Ethnopharmacology, Indigenous knowledge, Sexually transmitted disease

## Abstract

**Background:**

Sub-Saharan Africa faces one of the highest burdens of venereal diseases (VDs) globally. This review aims to critically evaluate the existing literature on the diverse Indigenous knowledge and medicinal plants utilised for treating VDs in sub-Saharan Africa.

**Methods:**

We used the Preferred Reporting Items for Systematic reviews and Meta-Analyses (PRISMA) protocol to guide the execution of the review. Relevant papers from scientific databases and search engines were assessed. The inclusion criteria included literature published from 2008 and May 16, 2024, and assessment of specific predetermined VDs. Medicinal plants based on certain ethnobotanical indices and data were recorded from each literature.

**Results:**

Among the 131 studies included in this review, a total of 20 relevant ethnobotanical reports were identified, with Nigeria and South Africa having the highest contributions (25% each). A high diversity and richness of 445 ethnobotanically valued anti-venereal plants (99 families) from over 872 Indigenous knowledge holders were inventoried. Plants with the highest diversity of use in traditional treatment of VDs are *Cassia abbreviata*, *Ziziphus mucronata*, *Ximenia caffra*, *Catharanthus roseus*, and *Terminalia prunioides*. The most represented families are Fabaceae (15.8%), Cucurbitaceae (5.9%), Solanaceae (5.9%), Euphorbiaceae (5%), and Combretaceae (5%). Roots and leaves were highly utilised with frequencies of 41.5% and 26.3%, respectively. The most used method of preparation are decoctions (36.7%) and infusions (12.2%), whereas oral route (72.9%) dominated the mode of administration of the medicinal plants.

**Conclusions:**

This review consolidated data from sub-Saharan Africa—notwithstanding a limited number of studies in quantitative synthesis—and identified a diverse array of ethnobotanically valued anti-venereal plants, enabling meaningful conclusions to be drawn for future ethnopharmacological assessments. Effective plant conservation and advancement of ethnobotanical research in the region require stringent regulations and cross-country collaborations.

**Supplementary Information:**

The online version contains supplementary material available at 10.1186/s41182-024-00651-y.

## Background

The imperativeness of managing infectious diseases cannot be over-emphasised as an estimated 15% global mortality rate is directly linked to infectious diseases annually [1]. This urgency is further heightened by the escalating prevalence of emerging diseases, which are being fuelled by increased globalisation, the emergence of multidrug-resistant pathogens, and the expanding reach of tropical and vector-borne diseases due to climate change. These factors are collectively putting an ever-increasing number of people at risk of life-threatening acute or chronic infections [1–3]. Notable among these infections are venereal diseases (VDs), which account for more than a million daily incidence globally with the majority being asymptomatic, resulting in a risk of passing the infection on to others [4]. Venereal diseases are infections that are spread by sexual activity, especially vaginal intercourse, anal and oral sex. The various categories of VDs with high prevalence and significant public health impact according to World Health Organisation [5] and Pfizer [6] include bacterial (chlamydia, gonorrhoea, and syphilis), viral (genital herpes, HIV/AIDS, and genital warts), and parasitic (trichomoniasis). The severity of these diseases is such that some of them can cause serious complications leading to infertility, and even death [4]. In accordance with the latest data available, the World Health Organization [7] report remains the most recent publication addressing the prevalence of VDs in low- and middle-income countries, particularly sub-Saharan Africa, which was identified as bearing approximately 40% of the global burden.

Even though conventional medicines are efficacious in the management of VDs, their shortcomings such as high cost of treatment, antibiotic resistance, and limited antimicrobial agents are undeniable, which often make patients find recourse to the ancient system of health care—traditional medicine [5, 8]. Contemporarily, ethnobotany is key to traditional medicine (TM), and important for understanding the relationship between TM practices, the conservation of medicinal plants and as an underpinning for scientific investigation of medicinal plants used for particular therapeutic purposes [9–18]. In line with this, a study conducted by Gbaranor et al. [19] on the treatment choices for sexually transmitted infections among 260 males in the South-South rural areas of Nigeria provides a compelling case. The study found that about 80% of the participants engaged in unprotected sex, while approximately 70% contracted a VD at some point. Remarkably, an overwhelming majority, 96.2%, expressed a preference for herbal medicine over antibiotics as their treatment of choice, which underscores the importance of Indigenous knowledge in disease management, particularly in Africa. This knowledge, often passed down through generations via oral tradition, is at risk of being lost due to inadequate documentation [2, 20]. Hence, by documenting and analysing the Indigenous uses of medicinal plants, ethnobotanical studies could potentiate advances in the development of new drugs and treatments to combat life-threatening diseases while also helping to preserve the rich cultural heritage associated with TM practices in Africa.

For centuries, VDs have been treated in Africa using plants, and such plants may face various conservation concerns in Indigenous communities where these resources are highly valued and utilised [21–25]. Ethnobotanical survey of medicinal plants used for the treatment of VDs may reveal a high diversity and richness of plant species and Indigenous knowledge systems among local communities, and may provide valuable information for the conservation and utilisation of these resources. Moreover, Indigenous cultures have a rich tradition of using certain plants for the treatment of several infectious diseases [26–28]. These practices may be adapted and integrated into modern medical practices to provide effective and culturally sensitive treatment options for VDs. This review is aimed at investigating the diverse medicinal plants used among local communities for the management of VDs across various ethnic regions of sub-Saharan African countries. It is also pertinent to identify potential threats to these plants and propose conservation strategies. Furthermore, a comparison of Indigenous knowledge techniques related to the management of VDs with modern medical practices, considering factors, such as dosage and toxicity, is crucial. This could pave the way for the adaptation and integration of these traditional and contemporary healthcare models. The review is delineated further to ascertain the bioprospecting potential of anti-venereal plants while identifying the research gaps in ethnobotany and ethnopharmacology.

## Methodology

**Fig. 1:**
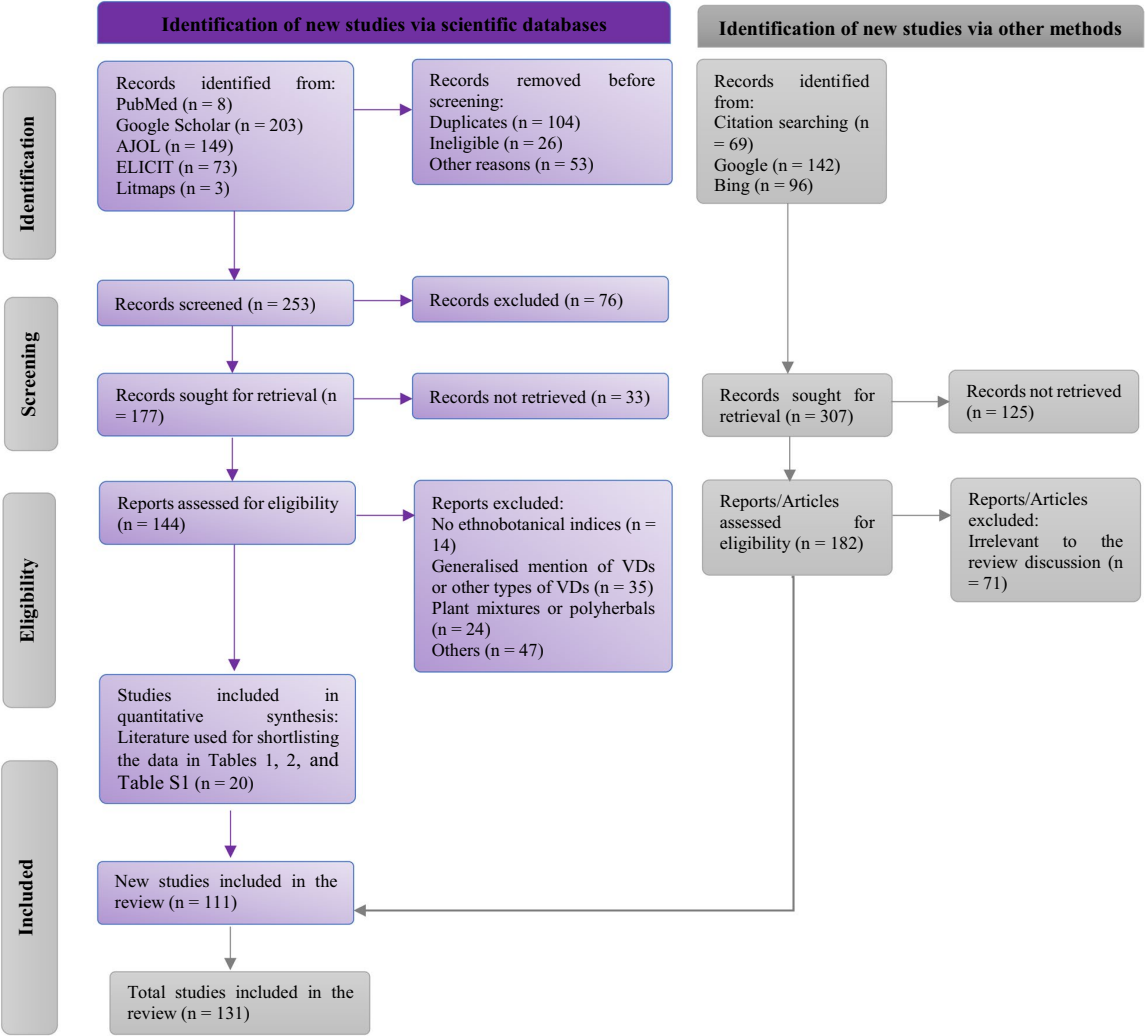
Flow chart of the systematic review protocol applied for inclusion of literature on Indigenous knowledge and plants used for treating venereal diseases in sub-Saharan Africa

This review was executed and guided by the recommendations in the Preferred Reporting Items for Systematic Reviews and Meta-Analyses (PRISMA) 2020 statement [29]. The flow chart of the review protocol is presented in Fig. 1.

### Literature search strategy

The time span entailed all the articles published not earlier than 15 years from when the literature search process of this review was completed (from 2008 and May 6, 2024 for the ethnobotanical study review), while a decade consideration was given for the ethnopharmacological aspect (from 2013 to May 16, 2024). The search terms such as “ethnobotanical survey,” “sexually transmitted diseases,” “African,” “venereal diseases,” “Indigenous knowledge,” among others were used both singly and in diverse combinations to systematically assess relevant papers from scientific databases including PubMed, Google Scholar, and AJOL. For example, the AND operator was used between certain terms to narrow the search to studies that covered overlapping themes, while OR operator was used between terms such as ‘sexually transmitted diseases’ and ‘venereal diseases’ to capture variations in terminology across different studies. Using Elicit (elicit.com) search tool, some research questions, “How can the ethnobotanical survey of medicinal plants used for the treatment of venereal diseases contribute to the preservation and enhancement of Indigenous knowledge and techniques? How have Indigenous cultures traditionally used medicinal plants for the treatment of venereal diseases? And how can these practices be adapted in modern times?” were used to find more relevant papers and supplement the literature. Litmaps, Google, and Bing were finally utilised to visualise and identify additional relevant papers. This process was continued until no further unobtained papers could be identified.

### Study selection

#### Exclusion criteria

Study titles and abstracts were screened manually to exclude the ones that are not related to the focus of this review. Articles pertaining to the ethnobotanical survey of generalised mention of VDs (no specificity) or other types of VDs (bacterial vaginosis, staphylococcus, genital ulcer and chancroid), binary or polyherbal mixture for the treatment of VDs, and countries outside Africa were all excluded from this review. Ethnobotanical studies that focus on HIV/AIDS opportunistic infections (tuberculosis, candidiasis, pneumonia, meningitis, herpes zoster, and Kaposi’s sarcoma) were also excluded.

#### Inclusion criteria

Relevant articles  were examined to determine their consonance with the eligibility criteria of this review. The specific inclusion criteria include the assessment of at least one of the ethnobotanical indices (fidelity level, informant consensus factor and use-value) and citation index (frequency of mention or number of citation); and articles published not earlier than 15 years from 2008 till date (May 6, 2024, when the ethnobotanical aspect of the review, i.e., records identification process for quantitative synthesis, was concluded). Finally, ethnobotanical studies relating to specific VDs (i.e., gonorrhoea, syphilis, chlamydia, trichomoniasis, genital herpes, genital warts, and HIV) were selected. Notably, a particular paper was considered for inclusion in the study despite lacking ethnobotanical indices and a citation index. This consideration was due to the paper’s definite mention of Indigenous knowledge and techniques for treating a certain VD (genital herpes) rarely found in other selected papers.

### Data retrieval/extraction

The selected studies were summarised and grouped into three main themes/categories. Moreover, from each study, medicinal plants used to treat VDs were listed. These plants were chosen based on their cultural significance, as determined by ethnobotanical citation indices such as fidelity level, informant consensus factor, use-value, frequency of mention, and number of citations. For each VD documented in the selected studies, about three plant species corresponding to their Indigenous management were extracted and tabulated in a Microsoft Excel spreadsheet. The categories used for tabulation included: family name, botanical name, vernacular name in the ethnic region, country of study, plant part(s) used, method of preparation, mode of administration, and traditional dosage. Beyond local importance, the cross-regional value of the medicinal plants were also enumerated based on their use across different regions and countries in sub-Saharan Africa. All botanical names were cross-verified with the World Flora Online database (www.worldfloraonline.org). Any plant that was not listed in this database, referred to as ‘unindexed’ subsequently in this study, was further validated using the Medicinal Plant Names Services portal (http://mpns.kew.org/mpns-portal).

Finally, to ensure a comprehensive analysis, another review of papers published from 2013 to May 16, 2024 were conducted to assess the ethnopharmacological profiles of the important plant species highlighted in the ethnobotanical studies. Specifying the exact cutoff date was necessary to provide a clear point of reference for future reviews as these dates mark the completion of our literature search in both ethnobotanical and ethnopharmacological areas. For each plant species noted in the ethnopharmacological studies, an extraction of relevant data was systematically performed. This information was then organised in a Microsoft Excel spreadsheet, categorising each plant by its common name, the country where the study was conducted, the pharmacodynamic activity of the specific plant part used, and the venereal diseases treated. This categorisation allowed for a detailed comparison with corresponding ethnobotanical survey findings. The structured approach ensured that ethnopharmacological research gaps (the valuable plant species from anecdotal evidence requiring preliminary experimental screening and further scientific scrutiny) were effectively identified.

### Data analysis

Microsoft Excel 2013 was used to organise the data obtained from the search, and to analyse the frequency distribution of families, plants, parts used, method of preparation, and mode of administration. In addition, the distribution of plants and families across the various VDs was analysed.

## Results

**Fig. 2:**
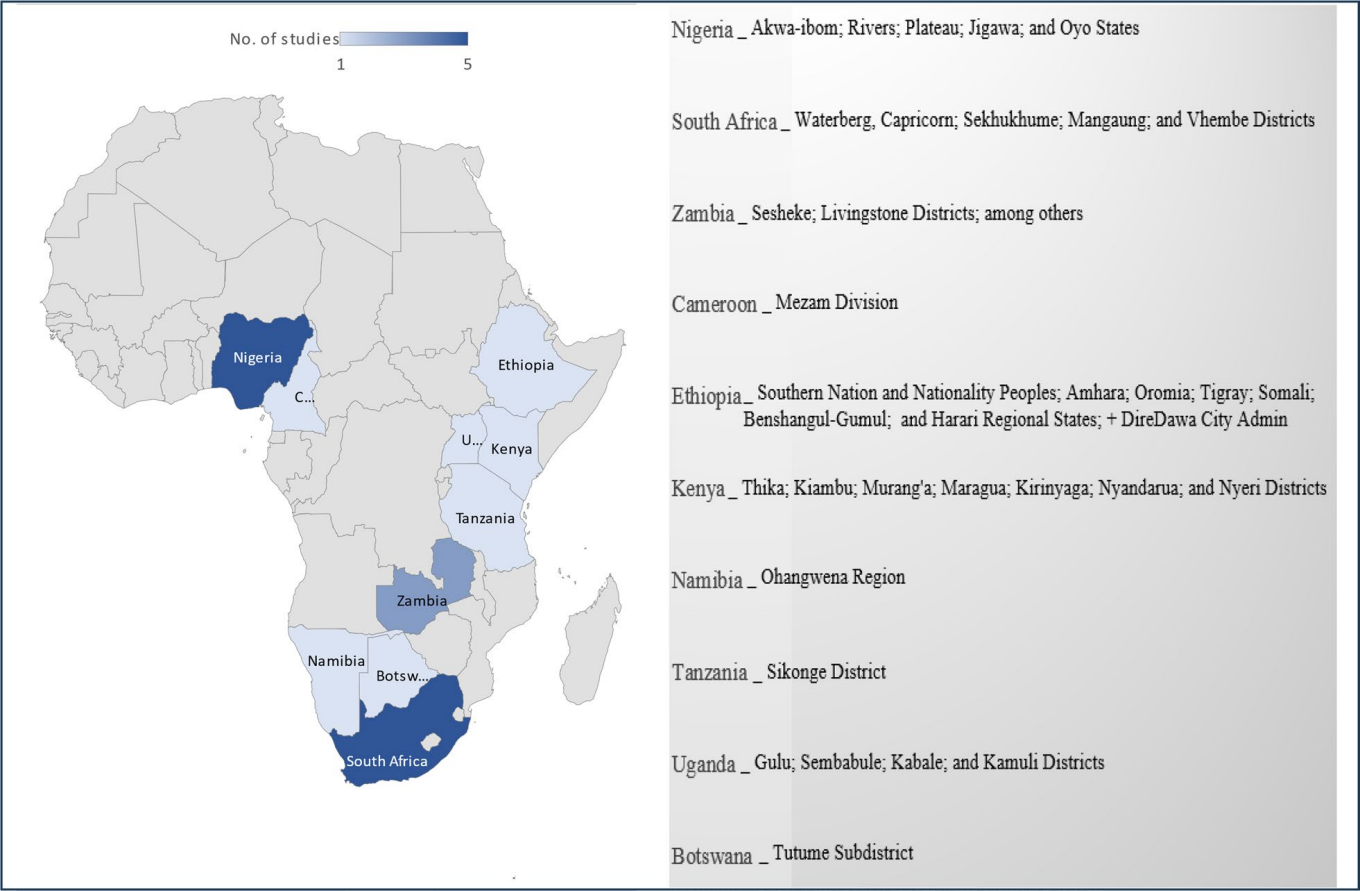
Heat map of sub-Saharan African countries/regions represented by ethnobotanical studies contributed to this review

Figure 2 presents an overview of countries contributing the recorded studies on the use of plants for venereal diseases (VDs). Nigeria and South Africa contribute the highest number of studies (50%) emanating from five different states (across three geopolitical zones) and five different municipalities (across two provinces), respectively, while Zambia represents three studies from five different provinces. Although the whole of Africa is primarily divided into five regions, only four regions constitute sub-Saharan Africa. As shown in Fig. 2, the current study is representative of all the regions in sub-Saharan Africa as it reviews ethnobotanical studies on VDs in Nigeria (West Africa); Cameroon (Central Africa); Ethiopia, Kenya, Uganda, and Tanzania (East Africa); and  Namibia, Botswana, South Africa and Zambia (Southern Africa).

This review revealed a high diversity and richness of medicinal plant species, ranging from 18 to 103 plant species in each selected study, and 445 plant species (belonging to 99 families) from over 872 Indigenous knowledge holders including TMPs and clerics (Table 1, Supplementary Table S1), used for treating VDs among communities in various countries/regions in sub-Saharan Africa.

**Table 1:** Ethnobotanical knowledge and Indigenous practices relating to the treatment of venereal diseases (VD) across some sub-Saharan African countries as reviewed from literature published from 2008 to May 6, 2024

Ethnobotanical studies	Themes/categories		
	Diversity of medicinal plant species	Indigenous knowledge and techniques	Conservation and utilisation status
Ajibesin et al. [30]	Reported 36 plant species from 26 families used to treat VDs including gonorrhoea and syphilis by TMPs in the Niger-Delta Region (Akwa-Ibom and Rivers States) of Nigeria	Documented the traditional knowledge of 100 traditional medicine practitioners (TMPs) in the Niger-Delta Region of Nigeria on medicinal plants as well as the information on local names, plant part used, therapeutic effect, diseases treated, method of preparation, and method of administration, dosage and duration of treatment	Determined certain ethnobotanical indices such as use-value (UV) and fidelity level (FL) of each plant species, and highlighted the importance of documenting traditional knowledge on medicinal plants before it disappears due to lack of documentation and loss of forest regions
Bizuayehu and Garedew [31]	Reported 100 anti-gonorrhoeal plant species belonging to 80 genera and 46 families in Southern Nation and Nationality Peoples, Amhara, Oromia, Tigray, Somali, Benshangul-Gumuz, and Harari Regional States, including Diredawa City Administration, Ethiopia	Documented the traditional knowledge of medicinal plants used for the treatment of gonorrhoea in Ethiopia, including their scientific and local names, habit of the plant, medicinal parts used, mode of preparation and route of administration	Determined no ethnobotanical indices, and also provided no information on the availability and threats of plant species nor suggested any conservation strategies, but indicated the frequency of citation for each plant species
Chinsembu [32]	Reported 52 plant species found in 25 families and 43 genera that were utilised in the management of some VDs in Sesheke District of Western Province, Zambia	Documented the traditional knowledge of medicinal plants that alleviate symptoms of VDs, including plant vernacular names, parts used, mode of preparation and administration in Sesheke District, Western Province, Zambia. Ethnobotanical data were collected from 20 traditional healers that manage patients presenting with VDs using semi-structured interviews and questionnaires	Assessed no ethnobotanical indices, and no information on the availability and threats of plant species nor suggested any conservation strategies, but highlighted the frequency index of each plant species
Chinsembu [33]	Reported 94 plant species from 39 families used by various knowledge holders to manage HIV/AIDS-related diseases in Livingstone District, Southern Province, Zambia	Documented traditional knowledge of 30 participants including 10 traditional healers about vernacular names, ethnomedicinal uses, parts used, preparation methods, and administration routes of plant remedies	Provided no information on the availability and threats of plant species or suggested any conservation strategies, but determined certain ethnobotanical index such as factor informant consensus and frequency index
Erasmus et al. [34]	Reported 18 plant species used by Bapedi traditional healers to treat gonorrhoea in Waterberg, Capricorn, and Sekhukhume Districts, Limpopo Province, South Africa	Documented the traditional knowledge of 30 Bapedi traditional healers, including the use of plants for medicine, plant parts used, method of preparation, and prescription	Determined no ethnobotanical indices nor suggested any conservation strategies, but highlighted the percentage frequency of quotation, and mentioned that both *Catharanthus roseus* and *Aloe marlothii* subsp. *marlothii* occur abundantly throughout the province and are currently not threatened
Gbadamosi and Egunyomi [35]	Reported 65 plant species of plants belonging to 38 families as remedies for the treatment and management of some VDs such as syphilis and HIV in Ibadan, Oyo State, Nigeria	Documented traditional knowledge from 300 respondents (TMPs, elderly individuals, clerics, and others) about vernacular names, parts used, ailment treated, method of preparation, mode of uses, dosage and duration of treatment of plant remedies	Determined no ethnobotanical indices and reported no availability and threats of plant species nor any conservation strategies, but noted the frequency of mention
Hedimbi and Chinsembu [36]	Reported 34 plants belonging to 19 different families that were used to manage various opportunistic infections related to HIV/AIDS in several villages of Ohangwena Region, northern Namibia, Namibia	Documented, from 28 knowledge holders, the traditional knowledge of people in Ohangwena region, including the use of plants for medicine, plant parts used, and disease conditions treated with the plants	Determined no ethnobotanical indices nor suggested any conservation strategies, but frequency of use for each plant species, and mentioned that destructive harvesting of plants should be prevented
Kacholi and Mvungi [37]	Reported 28 medicinal plants from 16 families used by Nyamwezi traditional health practitioners in managing VDs in Sikonge District, Tanzania	Documented the traditional knowledge of 23 TMPs about vernacular names, ethnomedicinal uses, parts used, preparation methods, dosage forms, administration routes and disease categories of plant remedies	Determined no ethnobotanical indices, but highlighted percentage citation index for each plant species, and suggested conservation strategies such as pharmacological investigations of the reported plants, provision of awareness to national traditional health practitioners (NTHPs) on sustainable harvest and conservation of the plants, and mentorship to the younger generation in an effort to preserve the Indigenous knowledge
Lamorde et al. [38]	Reported 103 medicinal plants used by traditional medicine practitioners for the treatment of HIV/AIDS and related conditions in four rural districts (Gulu, Sembabule, Kabale, and Kamuli) of Uganda	Documented, from 25 TMPs, the traditional knowledge, attitudes and practices related to HIV/AIDS recognition, control and treatment as well as the methods of preparation and administration of traditional medicine	Determined certain ethnobotanical index, such as informant consensus factor as well as frequency of mention by traditional medicine practitioners, but discussed no availability or threats to plant species nor any conservation strategies
Maema et al. [39]	Reported that 14 invasive alien species belonging to 10 families were used for the treatment of seven VDs in Waterberg district of Limpopo Province, South Africa	Documented, from 30 TMPs, the ethnobotanical applications of invasive alien species in the treatment of VDs, including plant parts used, preparation methods, administration mode, dosage and duration of treatment	Determined certain ethnobotanical indices, such as number of use report, fidelity level, and use value for each plant species, but reported no data on the availability and threats of plant species nor any conservation strategies
Mbambala et al. [40]	Reported 38 invasive alien plant species belonging to 23 families used in the treatment of HIV/AIDS-related symptoms by traditional healers of Vhembe District Municipality, Limpopo Province, South Africa	Documented the traditional knowledge of 21 traditional healers in Vhembe Municipality, including, parts used, common and local names of the plant species	Had no ethnobotanical indices nor any specific conservation strategies, but noted the availability of plant species and the frequency of use among traditional healers
Njoroge and Bussmann [41]	Reported 49 plant species in 30 families used in managing various VDs and reproductive health conditions in Thika, Kiambu, Murang’a, Maragwa, Kirinyaga, Nyandarua, and Nyeri Districts, Central Province of Kenya	Documented the traditional knowledge of resource group including males and females who depended on plant resources for managing VDs and reproductive health in 7 districts, including plant species, parts and methods of application	Determined no ethnobotanical indices, but provided the frequency of mention and informant consensus of each plant species, noting that *Warburgia ugandensis* Sprague and *Prunus africana* (Hook.f.) Kalkm. are already over-utilised and facing threat in Kenya need conservation measures
Ngobeni et al. [42]	Identified 35 plant species used for treating sexually transmitted infections (STIs) in Thaba ‘Nchu (Mangaung Metropolitan Municipality), Free State Province, South Africa	Documented information gathered through semi-structured interviews with 24 TMPs in Thaba ‘Nchu, South Africa. The study documented the traditional knowledge of medicinal plants, including local names, family names, plant parts used, companion plants, diseases treated, method of preparation, and mode of administration, and dosage	Determined the use value (UV) of the plant species and recommended the need for sustainable harvesting of plant materials
Noumi and Manga [43]	Reported 41 plant species belonging to 39 genera and 23 families used in the treatment of HIV/AIDS and its opportunistic infections in the population of Mezam Division, North–West Cameroon	Documented the traditional knowledge of preparations and administration method of plants used for treating HIV/AIDS among patients registered in the regional hospital of Bamenda based on 150 citations made by 25 informants, including traditional healers	Determined no ethnobotanical indices or any specific conservation strategies, but noted the percentage quotation of each plant species
Nyirenda and Chipuwa [44]	Reported 35 plant species from 20 families used for various medicinal purposes in Western, Copperbelt, Central, and Northern Provinces of Zambia	Documented the traditional knowledge of 20 primary informants (community elders) in Zambia, focusing on vernacular/local names of plants, plant parts used, medicinal uses, methods of preparation, and dosage	Emphasised the importance of documenting traditional knowledge on medicinal plants in Zambia to prevent the loss of valuable information and biodiversity. Ethnobotanical indices such as familiarity index (Fi) and relative frequency of citation (RFC) were calculated to assess the significance of each plant species
Ohemu et al. [45]	Reported 64 medicinal plants species, represented by 62 genera from 39 families used in the treatment of viral infections including HIV within Jos, Plateau State, Nigeria	Documented the traditional knowledge of over 30 participants (TMPs and some indigenes/residents) about plant vernacular names, parts used, mode of preparation, and administration through direct interviews using a structured questionnaire supported with a tape recorder and digital camera	Determined no ethnobotanical indices nor suggested specific conservation strategies, but highlighted the frequency of mention of each plant species
Omilani [46]	Reported 52 plants representing 34 families used in the treatment of VDs in Ibadan, Oyo State, Nigeria	Documented the traditional knowledge of TMPs (herb sellers, herbalists, and herbal therapists) about plant local names, parts used, method of preparation, dosage, and duration of treatment through direct interview using a well-structured questionnaire communicated in Yoruba	Determined no ethnobotanical indices nor any specific conservation strategies, but provided the frequency of mention, use mention index (UMi), and percentage UMi
Richard et al. [47]	Reported 83 plants from 38 taxonomical families used for managing VDs including HSV, HIV/AIDS, and other health conditions by TMPs in the Tutume Subdistrict, Central Botswana, Botswana	Documented the traditional knowledge of 13 TMPs in the Tutume subdistrict, Central Botswana, on medicinal plants, including local names, family names, plant parts used, diseases treated, methods of preparation, modes of administration, and dosage	Determined no ethnobotanical indices nor any conservation strategies
Salami et al. [48]	Reported 26 plant species from 15 families used in the treatment of VDs including gonorrhoea and syphilis in Dutse and Kiyawa LGAs, Jigawa State, Nigeria	Documented the traditional knowledge of medicinal plants by surveying 140 respondents in Tsilliya (Kiyawa) and Shuwarin markets (Dutse), Jigawa State, Nigeria. It included information on plant parts used, diseases treated, methods of preparation, and life form of plants	Recommended the need for conservation and sustainable use of medicinal plants to prevent its loss. It also mentioned the determination of frequency of citation for each plant species
Semenya et al. [49]	Reported 47 medicinal plant species belonging to 43 genera and 32 families used to treat some VDs in Waterberg, Capricorn, and Sekhukhune Districts, Limpopo Province, South Africa	Documented the traditional knowledge of 34 traditional healers, including plant vernacular names, parts used, dosage and duration of treatment via a semi-structured questionnaire, supplemented by field observations	Determined the percentage citation number but not the availability and threats of plant species used by Bapedi healers

**Fig. 3:**
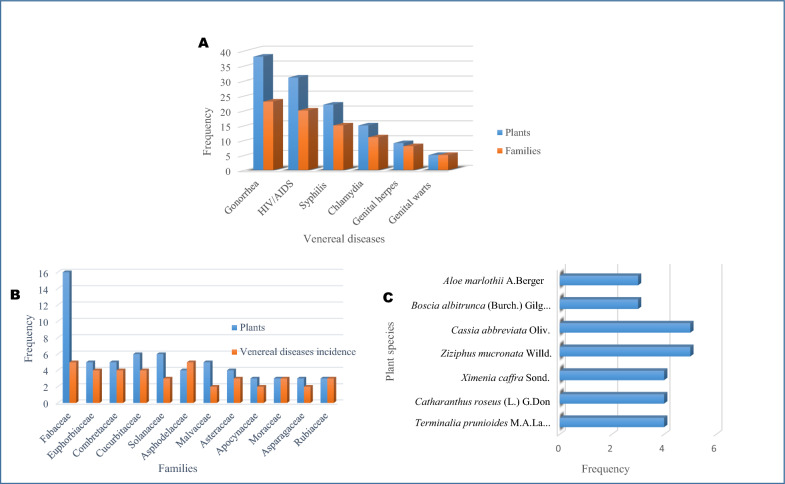
Distributon of **A** plants and families across various categories of venereal diseases; **B** families with three or more selected medicinal plants (*n* ≥ 3) associated with at least two (*n* ≥ 2) dstinct venereal disease treatments and **C** plant species with the highest diversity of use (*n* ≥ 3) among the selected medicinal plants for treating venereal diseases in sub-Saharan Africa

Summarily, 133 counts of various Indigenous knowledge and practices for the treatment of VDs were pooled from 20 ethnobotanical surveys across 10 sub-Saharan African countries (Tables 1 and 2). These counts are represented by the respective associated method of traditional preparation, administration, and posology for each mention of plants. A total of 98 plant species (including 1 unspecified plant species, 3 sub-species, and 1 variety) belonging to 43 families were recorded as remedies for various VDs. Specifically, 39 plant species (including 1 subspecies and 1 variety) belonging to 23 families are used in treating gonorrhoea; and 31 plant species (including 1 unspecified species and 1 variety) belonging to 20 families were indicated for HIV/AIDS management. In addition, 22 plant species belonging to 14 families were for syphilis; and 14 plant species (including 2 subspecies) belonging to 11 families for chlamydia (Fig. 3A).

**Table 2:** Plant species used singly for treating venereal diseases, shortlisted based on relatively high ethnobotanical index or frequency of citation,  and pooled from 20 published ethnobotanical studies conducted across various countries in sub-Saharan Africa

Family	Botanical name	Country	Vernacular name (Region/location)*	Part used	Preparation method	Mode of admin*	Dosage form	References
Indigenous knowledge and plants for treating gonorrhoea								
Amaryllidaceae	*Allium schoenoprasum* L	Nigeria	Aysoohia (Niger-Delta)	Leaves	Tincture	Topical	3 × 1 till recovery	Ajibesin et al. [30]
Anacardiaceae	*Anacardium occidentale* L	Nigeria	Cashew (Niger-Delta)	Leaves	Decoction	Topical	1 × 1 till recovery	
	*Lannea schweinfurthii* var. *stuhlmannii* (Engl.) Kokwaro; Synonym: *Lannea stuhlmannii* (Engl.) Eyles	Zambia	Mungangacha, Mucheche (Livingstone)	Roots and stem bark	Decoction	Oral and topical: The decoction of the crushed roots is drunk; stem bark decoction is used to wash affected skin	Not specified	Chinsembu [33]
Apiaceae	*Foeniculum vulgare* Mill	Ethiopia	Ensilal (Amharic), Kemona (Somali)	Leaves and root	Concoction, Crushing and decoction, Pounding	Oral	Not specified	Bizuayehu and Garedew [31]
Apocynaceae	*Acokanthera schimperi* (A.DC.) Benth. & Hook.f. ex Schweinf	Ethiopia	Merez (Amharic), Kararo (Oromo)	Leaves, root, root bark, stem bark, seed	Squeezing, Crushing and boiling, Chewing, Crushing and pounding, Infusion	Oral	Not specified	
	*Carissa spinarum* L	Ethiopia	Hagamsa (Oromo), Agam (Amharic)	Root bark and stem bark	Pounding, Crushing and boiling	Oral	Not specified	
	*Catharanthus roseus* (L.) G.Don	South Africa	Lepolomo le pinki la drop (Limpopo)	Roots	Decoction: The plant materials are cooked for 5–20 min	Oral	A cup (unspecified volume) of extracts taken 3 × 1 for 1 week	Erasmus et al. [34]
		South Africa	Unspecified local name (Waterberg)	Roots	Decoction: A handful of the roots are crushed and boiled for 15–20 min in 2 L water	Oral	Half cup (150 mL) is taken 3 × 1 till recovery	Maema et al. [39]
		South Africa	Lepolomo-le-le-pinki-la drop (Limpopo)	Roots	Decoction: The plant materials are boiled for 5–20 min	Oral	One tin cup of the extract is taken 3 × 1 till recovery	Semenya et al. [49]
Asphodelaceae	*Aloe marlothii* A.Berger	South Africa	Kgopha-ya-go-ema (Limpopo)	Roots	Decoction: The plant materials are boiled for 20 min	Oral	One tin cup of the extract is taken 3 × 1 till recovery	Semenya et al. [49]
	*Aloe marlothii* subsp. *marlothii*	South Africa	Sekgophasagoema (Limpopo)	Roots	Decoction: cooked for 20 min	Oral	A cup (unspecified volume) of extracts taken 3 × 1 for 1 week	Erasmus et al. [34]
	*Bulbine narcissifolia* Salm-Dyck	South Africa	Khomo-ea-balisa (Thaba ‘Nchu)	Leaves	Decoction	Oral	Not specified	Ngobeni et al. [42]
Asteraceae	*Dicoma anomala* Sond	South Africa	Hloenya (Thaba ‘Nchu)	Whole plant	Decoction	Oral	Not specified	Ngobeni et al. [42]
	*Sonchus oleraceus* L	Kenya	Mahiu (Central Province)	Roots	Decoction	Not specified	Not specified	Njoroge and Bussmann [41]
Cactaceae	*Opuntia ficus-indica* (L.) Mill	South Africa	Motloro (Limpopo)	Roots	Decoction: The plant materials are cooked for 20 min	Oral	A cup (unspecified volume) of extracts taken 3 × 1 for 1 week	Erasmus et al. [34]
		South Africa	Unspecified local name (Waterberg)	Roots	Decoction: The dried and crushed roots are boiled for 15–20 min in 2 L of water	Oral	Full cup (300 mL) is taken 2 × 1 till recovery	Maema et al. [39]
Combretaceae	*Combretum hereroense* Schinz	Zambia	Mububu (Western Province)	Leaves	Cold infusion	Oral	Not specified	Nyirenda and Chipuwa [44]
	*Terminalia prunioides* M.A.Lawson	Zambia	Muhonono (Sesheke)	Roots and leaves	Decoction. The plant materials are macerated together and boiled in water	Oral	Not specified	Chinsembu [32]
		Zambia	Mutala, Mukonono Mulumbu (Livingstone)	Roots	Infusion	Oral: Infusion of the dried blended outer parts of roots is drunk	Not specified	Chinsembu [33]
Cucurbitaceae	*Citrullus colocynthis* (L.) Schrad	Nigeria	Baara (Ibadan)	Fruit	Decoction and infusion: The plant material is diced into small pieces and the seeds removed, then boiled with potash and sieved	Oral	Three spoonsful of the decoction is taken till recovery	Omilani [46]
	*Citrullus lanatus* (Thunb.) Matsum. & Nakai	Nigeria	Baara (Ibadan)	Fruit	Infusion: The plant material is peeled, cut into small pieces, and soaked with 12 cubes of sugar in 1 L of cold water	Oral	250 mL of the solution is taken once daily after a meal	Gbadamosi and Egunyomi [35]
	*Citrullus naudinianus* Hook.f. Synonym: *Acanthosicyos naudinianus* (Sond.) Jeffrey	Namibia	Katangakamufifi (Ohangwena)	Fruit	Infusion	Oral	Not specified	Hedimbi and Chinsembu [36]
Euphorbiaceae	*Alchornea cordifolia* (Schumach..) Müll.Arg	Nigeria	Mbom (Niger-Delta)	Leaves	Crushed and juice applied	Oral	3 × 1 for 5 days	Ajibesin et al. [30]
	*Croton macrostachyus* Hochst. ex Delile	Ethiopia	Bisana (Amharic), Bakkannisa (Oromo), Asisi (Shinasha), Masinna (Sidama)	Leaves, Stem bark, Root	Squeezing, Powdering, Crushing and boiling, Cooking, Chewing, Crushing, Pounding and filtering	Oral	Not specified	Bizuayehu and Garedew [31]
	*Jatropha curcas* L	Nigeria	Unspecified local name (Dutse)	Roots	Decoction	Oral	Not specified	Salami et al. [48]
	*Ricinus communis* L	South Africa	Mupfure (Vhembe)	Whole plant, roots, fruit	Not specified	Not specified	Not specified	Mbambala et al. [40]
Fabaceae	*Cassia abbreviata* Oliv	Tanzania	Muzoka (Sikonge)	Roots and bark	Decoction	Oral	Not specified	Kacholi and Mvungi [37]
		Zambia	Mululwe (Livingstone)	Roots	Infusion	Oral: Root infusion is drunk	Not specified	Maema et al. [39]
	*Entada abyssinica* Steud. ex A.Rich	Tanzania	Mfutwamvula (Sikonge)	Roots	Decoction	Oral	Not specified	Kacholi and Mvungi [37]
	*Peltophorum africanum* Sond	Zambia	Munyele (Western Province)	Roots, stem bark, and leaves	Decoction: The plant materials are cut and boil in water	Oral	Not specified	Nyirenda and Chipuwa [44]
	*Senna alata* (L.) Roxb	Nigeria	Asunwon (Ibadan)	Leaves	Decoction: A decoction of the plant material is used to prepare food such as porridge	Oral	The herbal medicine is taken as food	Gbadamosi and Egunyomi [35]
	*Senna occidentalis* (L.) Link*;* Synonym: *Cassia occidentalis.* L	Zambia	Changu (Western Province)	Roots and stem bark	Decoction	Oral	Not specified	Nyirenda and Chipuwa [44]
Loganiaceae	*Strychnos cocculoides* Baker	Zambia	Muhuluhulu (Sesheke)	Roots	Infusion. The roots are crushed in water	Oral	Not specified	Chinsembu [32]
Meliaceae	*Melia azedarach* L	South Africa	Muserenga (Vhembe)	Whole plant, bark	Not specified	Not specified	Not specified	Mbambala et al. [40]
Moraceae	*Ficus polita* Vahl	Nigeria	Unspecified local name (Dutse)	Bark	Infusion	Oral	Not specified	Salami et al. [48]
Moringaceae	*Moringa oleifera* Lam	Nigeria	Unspecified local name (Dutse)	Roots	Decoction	Oral	Not specified	Salami et al. [48]
Musaceae	*Musa paradisiaca* L.; Synonym: *Musa sapientum* L	Kenya	Gituma kia irigu (Central Province)	Tuber	Infusion	Not specified	Not specified	Njoroge and Bussmann [41]
Myrtaceae	*Psidium guajava* L	South Africa	Unspecified (Waterberg)	Leaves	A tablespoon of the powdered plant material is infused in 2 L of hot water	Oral	Full cup (300 mL) is taken 2 × 1 till recovery	Maema et al. [39]
Olacaceae	*Ximenia caffra* Sond	Namibia	Ompeke (Ohangwena)	Leaves, stem	Decoction: The dried and crushed powder is boiled in water	Oral	Not specified	Hedimbi and Chinsembu [36]
		Zambia	Mulutulua (Sesheke)	Roots	Decoction	Oral	Not specified	Chinsembu [32]
		Tanzania	Munembwa (Sikonge)	Roots and leaves	Decoction	Oral	Not specified	Kacholi and Mvungi [37]
Papaveraceae	*Argemone ochroleuca* Sweet	South Africa	Zavhazavha (Vhembe)	Whole plant, roots, fruit	Not specified	Not specified	Not specified	Mbambala et al. [40]
Phytolaccaceae	*Phytolacca dodecandra* L'Hér	Ethiopia	Andode (Oromo), Endode (Amharic), Shebti (Tigrigna)	Leaves and Root	Crushing, decoction, powdering, pounding, squeezing, chewing, and concoction	Oral	Not specified	Bizuayehu and Garedew [31]
Poaceae	*Sporobolus pyramidalis* P. Beauv	Kenya	Kigutu/Kihato (Central Province)	Roots	Decoction	Not specified	Not specified	Njoroge and Bussmann [41]
Rhamnaceae	*Ziziphus mucronata* Willd	Namibia	Omukekete (Ohangwena)	Bark, leaves	Decoction	Oral	Not specified	Hedimbi and Chinsembu [36]
		South Africa	Mokgalo (Bapedi, Limpopo)	Roots	Decoction: The plant materials are boiled for 20 min	Oral	One tin cup of the extract is taken 3 × 1 till recovery	Semenya et al. [49]
Rubiaceae	*Pentanisia prunelloides* (Klotzsch) Walp	South Africa	Setima-mollo (Thaba ‘Nchu)	Whole plant	Decoction, Powdering	Oral, topical, and bath	Not specified	Ngobeni et al. [42]
Indigenous knowledge and plants for treating syphilis								
Arecaceae	*Elaeis guineensis* Jacq	Nigeria	Eyop (Niger-Delta)	Roots	Powder mixed with kernel oil	Oral	A cup (unspecified volume) of extracts is taken 3 × 1 till recovery	Ajibesin et al. [30]
Asparagaceae	*Agave sisalana* Perrine	South Africa	Unspecified (Waterberg)	Roots	Decoction: A handful of the plant materials are crushed and boiled for 15–20 min in 2 L of water	Oral	Full cup (300 mL) is taken 2 × 1 till recovery	Maema et al. [39]
Asteraceae	*Ageratum conyzoides* L	Nigeria	Imi-esu (Ibadan)	Whole plant, leaves	Tincture: A large quantity (3 kg) of the plant material is charred, and the powder is soaked in ethanol	Oral	One tot of the tincture is taken twice daily (2 × 1) after meals till recovery	Gbadamosi and Egunyomi [35]
	*Litogyne gariepina* (DC.) Anderb.; Synonym: *Epaltes alata* Steetz	Namibia	Odivadiva (Ohangwena)	Leaves	Ashing	Topical: Rubbing roasted leaves or powder into wounds	Not specified	Hedimbi and Chinsembu [36]
Bignoniaceae	*Kigelia africana* (Lam.) Benth	Zambia	Muzungula (Livingstone)	Stem bark and leaves	Decoction	Oral: Decoction of the stem bark and leaves is drunk	Not specified	Chinsembu [33]
			Umufungufungu, Muzungulwa, Muvungulwa, (Copperbelt)	Stem bark and leaves	Decoction, Exudate: Stem exudate is used as dressing for wounds/sores; decoction of crushed plant materials	Oral and topical application	Not specified	Nyirenda and Chipuwa [44]
Capparaceae	*Boscia albitrunca* (Burch.) Gilg & Benedict	Namibia	Omunghudi (Ohangwena)	Leaves and bark	Pounding and decoction	Oral and topical: The decoction extract is rubbed on the infected area and also ingested	Not specified	Hedimbi and Chinsembu [36]
		Zambia	Kabombwa–mutembwa (Western Province)	Root	Decoction	Oral	Not specified	Nyirenda and Chipuwa [44]
Combretaceae	*Terminalia kaiseriana* F.Hoffm	Tanzania	Muzima (Sikonge)	Roots	Decoction	Oral	Not specified	Kacholi and Mvungi [37]
	*Terminalia prunioides* M.A.Lawson	Zambia	Mutala, Mukonono, Mulumbu (Livingstone)	Roots	Infusion	Oral: Infusion of the dried blended outer parts of roots is drunk	Not specified	Chinsembu [33]
	*Terminalia sericea* Burch. ex DC	Zambia	Muhonono (Sesheke)	Roots, leaves	Maceration and decoction: The plant materials are macerated together and boiled in water	Oral	Not specified	Chinsembu [32]
Cucurbitaceae	*Momordica balsamina* L	Zambia	Lombwalombwa (Sesheke)	Whole plant	Decoction	Oral: The decoction is taken with porridge	Not specified	
Ebenaceae	*Diospyros lycioides* Desf	Zambia	Mupichu (Sesheke)	Leaves	Infusion: The plant materials are soaked in cold water for 3 days	Oral	Not specified	
Euphorbiaceae	*Euphorbia lateriflora* Schum. & Thonn	Nigeria	Enu opiri (Ibadan)	Stem	Powdering and concoction: The dried stem (peel) of the plant is powdered. A teaspoonful is added to eggs, then fried	Oral	The concoction is taken as food	Gbadamosi and Egunyomi [35]
Fabaceae	*Cassia abbreviata* Oliv	Tanzania	Muzoka (Sikonge)	Roots and bark	Decoction	Oral	Not specified	Kacholi and Mvungi [37]
	*Peltophorum africanum* Sond	Zambia	Munyele (Western Province)	Roots, stem bark, and leaves	Decoction: The plant materials are cut and boiled in water	Oral	Not specified	Nyirenda and Chipuwa [44]
	*Philenoptera cyanescens* (Schumach. & Thonn.) Roberty; Synonym: *Lonchocarpus cyanescens* (Schumach. & Thonn.) Benth	Nigeria	Nji (Niger-Delta)	Leaves and bark	Infusion	Oral	A cup (unspecified volume) of extracts is taken 3 × 1 till recovery	Ajibesin et al. [30]
	*Piliostigma reticulatum* (DC.) Hochst	Nigeria	Unspecified local name (Dutse)	Bark	Infusion	Oral	Not specified	Salami et al. [48]
Malvaceae	*Gossypium hirsutum* L	Nigeria	Ngobe (Niger-Delta)	Leaves	Decoction and infusion	Oral	A cup (unspecified volume) of extracts taken 3 × 1 for 10 days	
	*Waltheria indica* L	Namibia	Oshihakulamesho (Ohangwena)	Stem	Exudate	Topical: The sap is applied to wounds	Not specified	Hedimbi and Chinsembu [36]
Olacaceae	*Ximenia caffra* Sond	Tanzania	Munembwa (Sikonge)	Roots and leaves	Decoction	Oral	Not specified	Ajibesin et al. [30]
Rhamnaceae	*Ziziphus mucronata* Willd	Zambia	Muchechete, Mwichechete (Livingstone)	Fruit	Chewing	Oral and topical: The fruit is eaten raw, applied to wound, and put into porridge	Not specified	Chinsembu [33]
Solanaceae	*Solanum elaeagnifolium* Cav	South Africa	Unspecified (Waterberg)	Roots	Decoction: 4 pieces of roots are boiled for 15–20 min in 2 L of water	Oral	Full cup (300 mL) is taken 2 × 1 till recovery	Maema et al. [39]
	*Solanum mauritianum* Scop	South Africa	Unspecified (Waterberg)	Roots	Decoction: A handful of roots are boiled for 10–15 min in 2 L of water	Oral	Full cup (300 mL) is taken 2 × 1 till recovery	
Indigenous knowledge and plants for treating chlamydia								
Amaranthaceae	*Achyranthes aspera* L	Zambia	Tantajulo (Livingstone)	Roots, whole plant	Infusion and decoction	Oral and topical: The infusion of the roots or whole plant decoction is drunk; paste of plant is applied to affected skin	Not specified	Chinsembu [33]
Amaryllidaceae	*Gethyllis namaquensis* (Schönland) Oberm	South Africa	Naka tsa tholo (Bapedi, Limpopo)	Bulb	Maceration: The plant material is macerated in warm water for 24 h	Oral	One tin cup of the extract is taken 3 × 1 till recovery	Semenya et al. [49]
Asphodelaceae	*Aloe marlothii* A.Berger	South Africa	Kgopha-ya-go-ema (Bapedi, Limpopo)	Leaves	Decoction: The plant materials are boiled for 10 min	Oral	One tin cup of the warm extract is administered via a bulb syringe by healer once a day (1 × 1) till recovery	Semenya et al. [49]
Combretaceae	*Combretum hereroense* Schinz	Zambia	Mububu (Sesheke)	Leaves	Infusion	Oral	Not specified	Chinsembu [32]
Cucurbitaceae	*Cucumis myriocarpus* Naudin subsp. *myriocarpus*	South Africa	Magapyana (Bapedi, Limpopo)	Tuber	Decoction: The plant materials are boiled for 20 min	Oral	One tin cup of the extract is taken 3 × 1 till recovery	Semenya et al. [49]
Euphorbiaceae	*Ricinus communis* L	South Africa	Unspecified local name (Waterberg)	Roots, leaves	Decoction: The plant materials are crushed and boiled with 2 L water for 10–15 min	Oral	Full cup (300 mL) is taken 2 × 1 till recovery	Maema et al. [39]
Fabaceae	*Burkea africana* Hook	Zambia	Musheshe (Sesheke)	Roots and stem bark	Decoction: Dried plant parts are pounded into powder, boiled in water, and sieved	Enema: The filtrate is introduced into the urethra	Not specified	Chinsembu [32]
	*Cassia abreviata* Oliv	Tanzania	Muzoka (Tabora)	Roots, bark	Decoction	Oral	Not specified	Kacholi and Mvungi [37]
	*Senna didymobotrya* (Fresen.) H.S.Irwin & Barneby	South Africa	Unspecified local name (Waterberg)	Roots	Infusion and decoction: A tablespoon of powdered plant material is infused in 2 L of warm water or the roots are crushed and boiled for 10–15 min	Oral	Full cup (300 mL) is taken 2 × 1 till recovery	Maema et al. [39]
	*Vachellia nilotica* (L.) P.J.H.Hurter & Mabb.; Synonym: *Acacia nilotica* Delile	Zambia	Mukotokoto (Sesheke)	Leaves, roots, and stem bark	Pounding	Oral: The plant materials are pounded and mixed with warm water	Not specified	Chinsembu [32]
Moraceae	*Ficus sur* Forssk.; Synonym: *Ficus capensis* Thunb		Mukuyu (Livingstone)	Leaves	Decoction	Oral and topical: The fresh leaves are boiled in water and decoction is drank or used to wash warts and skin sores	Not specified	Chinsembu [33]
Proteaceae	*Protea caffra subsp. *caffra*; Synonym: *Protea caffra* Meisn	South Africa	Unspecified	Seeds	Pounding	Oral	Six teaspoons of the plant extract is taken in a cup of warm water 3 × 1 for a week	Semenya et al. [49]
Rhamnaceae	*Ziziphus mucronata* Willd	Tanzania	Kavogole (Sikonge)	Roots	Decoction	Oral	Not specified	Kacholi and Mvungi [37]
Rubiaceae	*Mitragyna inermis* (Willd.) Kuntze	Nigeria	Unspecified local name (Dutse)	Roots	Decoction	Oral	Not specified	Salami et al. [48]
Indigenous knowledge and plants used for treating genital herpes								
Asphodelaceae	*Aloe marlothii* A.Berger	Botswana	Gonde (Tutume)	Leaves	Decoction: Cut fresh leaves into small pieces. Measure the pieces in a full teaspoon and soak in 2 L cold water	Oral	3 × 1 till recovery	Richard et al. [47]
Bignoniaceae	*Kigelia africana* (Lam.) Benth	Zambia	Umufungufungu, Muzungulwa, Muvungulwa, (Copperbelt Province)	Stem bark and leaves	Decoction, Exudate: Stem exudate is used as dressing for wounds/sores; decoction of crushed plant materials	Oral and topical application	Not specified	Nyirenda and Chipuwa [44]
Ebenaceae	*Euclea divinorum* Hiern	Zambia	Musokola (Sesheke)	Stem and leaves	Decoction	Topical: The decoction is used to wash syphilitic ulcers	Not specified	Chinsembu [32]
		Zambia	Munyansyabweli (Livingstone)	Roots	Decoction	Oral: The decoction of the ground roots is drunk	Not specified	Chinsembu [33]
Fabaceae	*Abrus precatorius* L	Zambia	Musolosolo (Livingstone)	Whole plant	Decoction	Oral and topical: The decoction of the whole plant is drank	Not specified	
	*Phaseolus vulgaris* L	Botswana	Ipule (Tutume)	Seed	Pounding: The plant material is crushed while fresh to make a paste of the medicine	Topical: Paste and smear over sores/wounds	As needed till recovery	Richard et al. [47]
Hyacinthaceae	*Ledebouria cooperi* (Hook.f.) Jessop	Botswana	Phalalume (Tutume)	Root	Pounding and infusion, Ashing: Pound while fresh alongside other prescribed medicinal plants. Add burnt grass powder and mix in a 2 L of warm water bottle	Oral	3 × 1 till recovery	Richard et al. [47]
Menispermaceae	*Cissampelos mucronata* A.Rich	Zambia	ltende (Sesheke)	Roots and leaves	Infusion	Topical: The cold infusion is used as a dressing to heal wounds	Not specified	Chinsembu [32]
Phyllanthaceae	*Flueggea virosa* (Roxb. ex Willd.) Royle	Botswana	Nshangoma (Tutume)	Root	Decoction: Boil the roots in water then cool	Oral	2 × 1 till recovery	Richard et al. [47]
Rhamnaceae	*Ziziphus mucronata* Willd	Botswana	Ntjetjeni (Tutume)	Leaf	Pounding: The plant material is crushed while fresh to make a paste of the medicine	Topical: Paste and smear over the sore	As needed until the sore is healed	Richard et al. [47]
Vitaceae	*Ampelocissus obtusata* Planch	Zambia	Munsansa (Sesheke)	Roots	Infusion	Topical: The cold infusion is used as a dressing to heal wounds	Not specified	
Indigenous knowledge and plants for treating genital warts								
Apocynaceae	*Catharanthus roseus* (L.) G.Don	South Africa	Unspecified (Waterberg)	Roots	Decoction: A handful of the roots are crushed and boiled for 15–20 min in 2 L water	Oral	Half cup (150 mL) is taken 3 × 1 till recovery	Maema et al. [39]
Asphodelaceae	*Aloe arborescens* Mill	South Africa	Kgopha-ya-fase (Bapedi, Limpopo)	Roots	Decoction: The plant materials are boiled for 20 min	Oral	One tin cup of the extract is taken 3 × 1 till recovery	Semenya et al. [49]
Cactaceae	*Opuntia stricta* (Haw.) Haw	South Africa	Unspecified (Waterberg)	Roots	Decoction: A handful of the plant materials is crushed and boiled for 10–15 min in 2 L water	Oral	Full cup (300 mL) is taken 2 × 1 till recovery	Maema et al. [39]
Meliaceae	*Entandrophragma caudatum* Sprague	Zambia	Mupamena (Sesheke)	Roots and fruits	Decoction and ashing	Oral and topical: The roots are boiled and the solution is drunk. The fruit peels are burnt, mixed with vaseline and rubbed on genital warts	Not specified	Chinsembu [32]
Moraceae	*Ficus natalensis* Hochst	Zambia	Mutaba (Sesheke)	Leaves	Pounding	Topical: The plant materials are pounded and rubbed on the genital warts	Not specified	
Solanaceae	*Nicotiana glauca* Graham	South Africa	Unspecified (Waterberg)	Roots	Decoction: A handful of the plant materials is crushed and boiled for 10–15 min in 2 L water	Oral	Half cup (150 mL) is taken 2 × 1 till recovery	Maema et al. [39]
Indigenous knowledge and plants for treating HIV/AIDS								
Anacardiaceae	*Lannea schweinfurthii* var. *stuhlmannii* (Engl.) Kokwaro; Synonym: *Lannea stuhlmannii* (Engl.) Eyles	Zambia	Mungangacha, Mucheche (Livingstone)	Roots and stem bark	Decoction	Oral and topical: The decoction of the crushed roots is drunk	Not specified	Chinsembu [33]
Araliaceae	*Cussonia paniculata* Eckl. & Zeyh	South Africa	Mots’ets’e (Thaba ‘Nchu)	Root	Decoction	Oral	Not specified	Ngobeni et al. [42]
Asparagaceae	*Asparagus microraphis* Baker	South Africa	Lereratau (Thaba ‘Nchu)	Root	Decoction	Oral	Not specified	Ngobeni et al. [42]
Asphodelaceae	*Aloe arborescens* Mill	South Africa	Kgopha-ya-fase (Bapedi, Limpopo)	Roots	Decoction: The plant materials are boiled for 20 min	Oral	One tin cup of the extract is taken 3 × 1 till recovery	Semenya et al. [49]
	*Aloe schweinfurthii* Baker.; Synonym: *Aloe barteri* Baker	Cameroon	Aloe vera (Mezam)	Leaves	Crushing: 250 mL of the plant materials are crushed into paste and added to 250 mL of honey	Oral	Two spoonful of the solution is taken 30 min before the meal 3 × 1 till recovery	Noumi and Manga [43]
	*Aloe secundiflora* Engl	Kenya	Mugwanugu (Central Province)	Leaves	Decoction	Not specified	Not specified	Njoroge and Bussmann [41]
	*Aloe* spp.	Uganda	Kíkaka (Kamuli), Kigagi (Sembabule), Rukaka (Kabale), Ataka-rach (Gulu)	Leaves and root bark	Not specified	Not specified	Not specified	Lamorde et al. [38]
Asteraceae	*Ageratum conyzoides* L	Nigeria	Imi-esu (Ibadan)	Whole plant, leaf	Tincture: A large quantity (3 kg) of the plant material is charred, and the powder is soaked in ethanol	Oral	One tot of the tincture is taken twice daily (2 × 1) after meals till recovery	Gbadamosi and Egunyomi [35]
	*Dicoma anomala* Sond	South Africa	Hloenya (Thaba ‘Nchu)	Root	Decoction	Oral	Not specified	Ngobeni et al. [42]
Basellaceae	*Anredera cordifolia* (Ten.) Steenis	South Africa	Unspecified (Waterberg)	Aerial tuber	Decoction: A handful of the plant materials is crushed and boiled for 10–15 min in 2 L of water	Oral	Half cup (150 mL) is taken 3 × 1 till recovery	Maema et al. [39]
Burseraceae	*Boswellia dalzielii* Hutch	Nigeria	Ararabi (Jos)	Leaves and stem bark	Decoction and powder	Oral and topical	Not specified	Ohemu et al. [45]
Capparaceae	*Boscia albitrunca* (Burch.) Gilg & Benedict	Zambia	Kabombwa–mutemwa (Sesheke)	Roots	Decoction	Oral: The solution is drunk while warm	Not specified	Chinsembu [32]
			Kabombwa–mutembwa (Western Province)	Leaves	Decoction	Oral	Not specified	Nyirenda and Chipuwa [44]
	*Boscia salicifolia* Oliv	Zambia	Mulaba, Kabombwe (Livingstone)	Roots	Infusion	Oral: The infusion of the ground roots is drunk	Not specified	Chinsembu [33]
Combretaceae	*Terminalia hylodendron* (Mildbr.) Gere & Boatwr.; Synonym: *Pteleopsis hylodendron* Mildbr	Cameroon	Sikon (Mezam)	Stem bark	Pounding and decoction: 0.5 kg of the plant materials are pounded and boiled for 15 min in 3 L of water and then cooled	Oral and topical	One glassful of the decoction is drunk 2 × 1 till recovery. The decoction extract is also used to rub the body parts affected by herpes zoster and kaposi’s sarcoma, engendering quick and good result	Noumi and Manga [43]
	*Terminalia prunioides* M.A.Lawson	Zambia	Mutala, Mukonono, Mulumbu (Livingstone)	Roots	Infusion	Oral: Infusion of the dried blended outer parts of roots is drunk	Not specified	Chinsembu [33]
Cucurbitaceae	*Momordica balsamina* L	Zambia	Lombwalombwa (Sesheke)	Whole plant	Decoction	Oral: The decoction is taken with porridge	Not specified	Chinsembu [32]
Euphorbiaceae	*Jatropha curcas* L	Nigeria	Biydazogu (Jos)	Leaves and roots	Decoction or powder	Oral	Not specified	Ohemu et al. [45]
Fabaceae	*Cassia abbreviata* Oliv	Zambia	Mululwe (Sesheke)	Stem bark and roots	Maceration	Oral: The solution is drunk	Not specified	Chinsembu [32]
	*Erythrina abyssinica* DC	Uganda	Muyirikiti (Kamuli), Kiko (Kabale), Lucoro (Gulu)	Root wood and stem bark	Not specified	Not specified	Not specified	Lamorde et al. [38]
Hypoxidaceae	*Hypoxis hemerocallidea* Fisch., C.A.Mey. & Avé-Lall	South Africa	Titikwane/sesogadi (Bapedi, Limpopo)	Tuber	Pounding/decoction: The plant material is boiled for 20 min	Oral	Five teaspoons of the pounded extract is taken with soft porridge 3 × 1 till recovery; while one tin cup of the decoction extract is taken 3 × 1 till recovery	Semenya et al. [49]
Malvaceae	*Adansonia digitata* L	Tanzania	Mibuyu (Sikonge)	Stem bark	Infusion and maceration	Oral: The plant materials are infused in warm water and drunk	Not specified	Kacholi and Mvungi [37]
	*Bombax buonopozense* P.Beauv	Cameroon	Esodum (Mezam)	Stem bark	Pounding: 500 g of the pounded plant materials are mixed and homogenized	Oral	One glassful of the solution extract is taken morning and evening after the meal. Instruction: The bottle where the extract is preserved must be well closed	Noumi and Manga [43]
Phyllanthaceae	*Bridelia micrantha (*Hochst.) Baill	Kenya	Mukoigo (Central Province)	Bark	Decoction	Not specified	Not specified	Njoroge and Bussmann [41]
Rosaceae	*Prunus africana* (Hook.f.) Kalkman	Kenya	Muiri (Central Province)	Bark	Decoction	Not specified	Not specified	Njoroge and Bussmann [41]
Rubiaceae	*Sarcocephalus latifolius* (Sm.) E.A. Bruce	Uganda	Mutamatama (Kamuli), Munyu (Gulu)	Root bark and whole root	Not specified	Not specified	Not specified	Lamorde et al. [38]
Rutaceae	*Harrisonia abyssinica* Oliv	Tanzania	Msomwanjala (Sikonge)	Roots	Decoction	Oral	Not specified	Kacholi and Mvungi [37]
	*Zanthoxylum humile* (E.A.Bruce) P.G.Waterman	South Africa	Monokwane (Bapedi, Limpopo)	Roots	Decoction: The plant materials are boiled for 20 min	Oral	One tin cup of the extract is taken 3 × 1 till recovery	Semenya et al. [49]
	*Nicotiana tabacum* L	Nigeria	Taba (Ibadan)	Leaves	Infusion: The plant materials are soaked in cow bile	Oral	Two table spoonful of the extract are taken twice daily after meals	Gbadamosi and Egunyomi [35]
Solanaceae	*Solanum campylacanthum* Hochst. ex A.Rich.; Synonym: *Solanum panduriforme* Drège ex Dunal	Zambia	Ntulwantulwa (Western Province)	Roots	Infusion: Roots are cut and macerated in water	Oral	Not specified	Nyirenda and Chipuwa [44]
	*Solanum mauritianum* Scop	South Africa	Unspecified (Waterberg)	Roots	Decoction: A handful of roots are boiled for 10–15 min in 2 L of water	Oral	Full cup (300 mL) is taken 2 × 1 till recovery	Maema et al. [39]
Verbenaceae	*Lantana camara* L	South Africa	Unspecified (Waterberg)	Leaves, twigs	Decoction: A handful of the plant materials is crushed and boiled for 10–15 min in 2 L of water	Oral	Full cup (300 mL) is taken 2 × 1 till recovery	Maema et al. [39]

Mode of admin*: mode of administration | (Region)*: the area where the ethnobotanical survey was conducted | In the dosage form column, 1 × 1, 2 × 1, and 3 × 1 represent once daily, twice daily, and thrice daily, respectively | *Protea caffra subsp. *caffra*; Synonym: *Protea caffra* Meisn. could only be verified with the Medicinal Plant Names Services portal

In the analysis of the 98 recorded plants (Table 2), 12 families were identified as dominant, having at least 3 plant species and occurring across a minimum of 2 VDs (Fig. 3B). The Fabaceae family (15.8%) was the most dominant with 16 plant species, followed by Cucurbitaceae (5.9%), Solanaceae (5.9%), Euphorbiaceae (5%), Combretaceae (5%), and Malvaceae (5%). Notably, Fabaceae was involved in the management of 5 out of the 6 VDs (Table 2), with the exception of genital warts. Similarly, Asphodelaceae contributed to all treatments, excluding syphilis. Furthermore, Combretaceae, Cucurbitaceae, and Euphorbiaceae were associated with the management of 4 VDs, particularly gonorrhoea, syphilis, and HIV/AIDS.

Figure 3C depicts plant species with the highest diversity of use for treating VDs in Indigenous knowledge (IK) and practice across various regions/countries in sub-Saharan Africa, as observed in Table 2. For instance, *Cassia abbreviata* Oliv., with the highest frequency, is used in 3 different regions of Zambia and Tanzania for treating various VDs (gonorrhoea, syphilis, chlamydia, and HIV/AIDS), with some variations in IK applications. *Ziziphus mucronata* Willd. is also used for various VDs (gonorrhoea, syphilis, and chlamydia) in 5 different countries (Namibia, South Africa, Zambia, Botswana, and Tanzania), with major differences in the plant parts used. *Ximenia caffra* Sond. is used in 3 distinct regions and countries (Namibia, Zambia, and Tanzania) for syphilis and gonorrhoea, with some differences in IK practices (particularly, the plant parts used). Despite the closely similar IK practices, *Terminalia prunioides* M.A. Lawson is used in 3 distinct regions of Zambia as a remedy for gonorrhoea, syphilis, and HIV/AIDS.

**Fig. 4:**
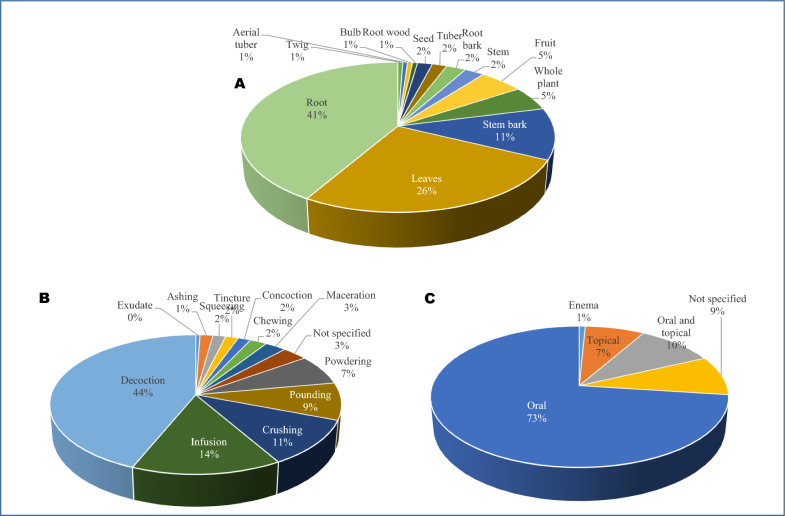
Percentage distribution of **A** parts used, **B** preparation methods, and **C** mode of administration of selected medicinal plant materials for treating venereal diseases in sub-Saharan Africa

Figure 4A illustrates the frequency of each plant part used across all VD treatment categories and various sub-Saharan African regions/countries. Root (41%) constitutes the highest frequency in the recorded plants. In some cases, certain plant parts of a species are used for various VD treatment categories in different regions/countries. For instance, *Cassia abbreviata* Oliv. root is used in Tanzania, Zambia, and South Africa, and the record of its frequency is taken for each country. Likewise, *Terminalia prunioides* M.A. Lawson root is used for the treatment of gonorrhoea, syphilis, and HIV/AIDS, and the account of its frequency is taken for each VD.

The 98 plants and their respective materials are prepared by various methods (Table 2). Figure 4B summarizes the frequency of occurrence of these methods of preparation. For instance, *Phytolacca dodecandra* L’Hér leaves and root are prepared by crushing, decoction, powdering, pounding, squeezing, chewing, and concoction for the treatment of gonorrhoea, and the frequency record is taken for each of the mentioned methods of preparation for the plant materials. Decoction as a method of plant material preparation (44%) constitutes the highest frequency, followed by infusion (14%) and crushing (11%).

Figure 4C depicts the distribution in terms of mode of administration of plant materials and preparations. The oral route (73%) is the most popular mode of administration, while an estimated 9% of the recorded plants were lacking (not specified in the primary source) a particular mode of administration.

**Fig. 5:**
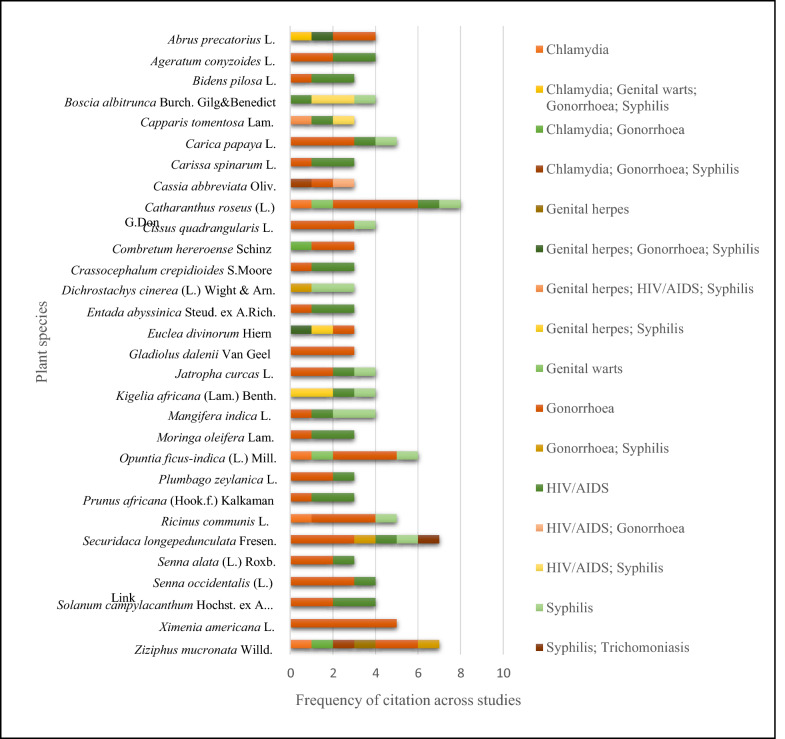
Medicinal plants with the most occurrence (*n* ≥ 3 ethnobotanical studies) for managing venereal diseases across different regions in sub-Saharan Africa

The top 30 frequently cited medicinal plant species used for the treatment of different venereal diseases across various regions in sub-Saharan Africa are indicated in Fig. 5. These plants were ranked based on their citation in 3 or more studies, out of a total of 445 plant species listed in Supplementary Table S1. This compilation pools data from 20 ethnobotanical surveys conducted in 10 sub-Saharan African countries (Fig. 2), as assessed in this review. For instance, the highest number of use-report (8) for VDs covering 3 or more studies was observed for *Catharanthus roseus* (L.) G.Don, cited by four different studies across three regions (Limpopo, Waterberg, and Vhembe) in South Africa. This was followed by *Ziziphus mucronata* Willd. and *Securidaca longepedunculata* Fresen., which were each cited seven times for the treatment of VDs and each documented by six different studies across five countries, including Botswana, South Africa, Namibia, Tanzania, Nigeria, Ethiopia, Uganda, and Zambia. Other cross-regionally important species in Fig. 5 include *Opuntia ficus-indica* (L.) Mill., *Carica papaya* L., *Ricinus communis* L., and *Ximenia americana* L., each cited in at least three ethnobotanical studies.

## Discussion

All the reviewed papers used rigorous methodologies such as structured or semi-structured questionnaires, interviews, and statistical analyses. However, there are some differences among the papers in terms of their specific methods (Table 1). All the reviewed papers, other than Maema et al. [39] and Salami et al. [48], mentioned the specific local names of each medicinal plant species. It is possible that the medicinal plant species studied are naturalised and not endemic to the study area as observed by Maema et al. [39] or a complete omission by Salami et al. [48]. Two studies included in this review showed distinctiveness from all other studies in that they provided a broader ethnobotanical knowledge of anti-venereal plants [31, 38].

### Venereal diseases in Sub-Saharan Africa

Sub-Saharan Africa accounts for the highest (about 40%) of the global burden of curable and non-curable VDs, with an incidence of 240 per 1000 persons and more than 93 million annual incidence [7, 50–52]. As of 2021, there were a total of 23.1 million people in West, Central, East, and Southern Africa living with HIV [53, 54]. In the same year, South Africa and Nigeria, from Southern and West Africa, respectively, recorded the highest mortality due to AIDS globally, with around 51,000 deaths each [55]. This likely explains the high proportion of studies contributed from these two countries to this review (Fig. 2). Corroboratively, Wand et al. [56] reported that South Africa had the highest burden of VDs globally. On the other hand, accurate national data on the incidence and prevalence of distinctive VDs is not available in the other 8 countries represented in this review (Fig. 2). However, some epidemiological investigations have shown that VDs are a serious public health problem in these countries [51, 57–66]. According to the classification by the World Bank (https://openknowledge.worldbank.org/pages/focus-sub-saharan-africa) and the ISS African Futures (https://futures.issafrica.org/geographic/regions/sub-saharan-africa/), there are 48 countries in sub-Saharan Africa. Thus, approximately one-fifth of these countries are represented in this review. Therefore, there is a need for comprehensive ethnobotanical studies on VDs across the whole region, considering the high burden of these diseases. With the provision of research funding, scientific collaboration, and networks, ethnobotanical knowledge can be made available across all sub-Saharan African countries as a potential source of information or resources for bioprospecting studies aimed at curtailing the burden of VDs.

### Diversity and richness of plant species

Many of the ethnobotanical studies documented a wide variety of medicinal plant species used for treating VDs. Several studies, for example, mentioned the use of plants from the Fabaceae and Combretaceae families, occurring more frequently than others [32, 33, 35–37, 42, 44, 46]. Fabaceae, Euphorbiaceae, Combretaceae, and Cucurbitaceae being the most important documented source of anti-venereal plants in sub-Saharan Africa (Fig. 3B), is in consonance with the extensive review by Van Wyk [67], which showed that these plant families rank 1st, 5th, 6th, and 9th, respectively, of the over-used plant families in African traditional medicine. Moreover, due to the limitation of ethnobotanical indices, i.e., the lack of a consistent measure for the probability distribution, which precludes them from being juxtaposed with other studies [15, 68], it became necessary, in addition to the 98 recorded plant taxa selected based on their ethnobotanical importance in each of the 20 quantitative studies (Table 2), to outline the most ethnobotanically valued anti-venereal ethnomedicine across sub-Saharan Africa (Fig. 5) from the documented 445 plants species (Supplementary Table S1). The consistent use of these plants across different regions and countries (Fig. 5) underscores their perceived efficacy and extensive importance. Furthermore, these findings could foster cross-regional cooperation, such as the sharing of knowledge and resources, in further anti-venereal studies and the future development of traditional medicines in sub-Saharan Africa. On the other hand, it is interesting to note that various countries/regions in sub-Saharan Africa share similar Indigenous knowledge about medicinal plants for VDs. However, the Indigenous knowledge and techniques employed in the application of these plants often vary across the regions (Table 2).

### Traditional uses, preparation and administration methods

There are some parallelism and distinctiveness in the specific details of the Indigenous knowledge and techniques among the selected studies. For instance, all the selected papers noted at least one plant part involved in the ethnobotanical uses for each VD investigated. Furthermore, most of the eligible papers documented specific modes of preparation and administration of medicinal plants used for the treatment of VDs (Table 2), except for only six studies [30, 31, 37, 41, 42, 48], which only provided general information of these methods. However, Njoroge and Bussmann [41] made available a specific method of preparation only, while some papers failed to specify the method of preparation and mode of administration [38, 40]. Indigenous communities hold vast knowledge of medicinal plants and majorly use decoction, infusion, and powdering methods for the preparation of traditional medicine [12, 69–71]. This is in agreement with the findings of this review (Fig. 4B). Finally, Ajibesin et al. [30] and Crane et al. [72] noted that it is important to preserve Indigenous knowledge before it disappears due to the current limited documentation.

### Conservation and utilisation status of ethnobotanically valued anti-venereal plants

The conservation and utilisation status of medicinal plants may vary depending on several factors such as their use value, fidelity level, relative frequency of citation, and informant consensus factor [68, 73, 74]. These factors can help understand how different communities value and use medicinal plants and can provide insights into which plants are most important for conservation and sustainable utilisation efforts [75, 76]. Hence, the recorded plant species in Table 2 could be considered for cultivation and conservation for the fact that they were selected based on their local importance and high ethnobotanical indices in the study areas/regions. Although the concern of traditional healers in sub-Saharan Africa about the qualitative inferiority of cultivated medicinal plant species over wild-grown species has, hitherto, curtailed the domestication of medicinal plants over time, phytomedicine scientists have made efforts to debunk the idea by documenting the experimented variations [24, 77]. Many of these studies showed higher therapeutic potential of cultivated plants compared to wild-grown plants, with all investigated cultivated species exhibiting a promising alternative for significant active metabolites yield [78–85]. Moreover, there seems to be a paradigm shift among many traditional healers with many of them accepting the use of cultivated medicinal plants or cultivating the plants themselves [86].

#### Availability and threats

None of the quantitative studies in this review, except Mbambala et al. [40], documented the availability and threats of their listed plant species. This may affect the accuracy and reliability of the utilisation status and conservation discussion on the plants. Although several papers in this review mentioned the need for conservation strategies to ensure the sustainable use of medicinal plants, they did not provide specific information on the availability and threats of these plant species, other than Mbambala et al. [40]. The authors indicated that none of its investigated anti-venereal plants have low availability status. Therefore, further research is needed to better understand the availability and threats of these plants and to develop effective conservation strategies.

#### Conservation strategies

Succinct examples of conservation strategies were documented in a study by Semenya et al. [87], which was excluded from Table 1 due to its overlapping Indigenous knowledge data with Semenya et al. [49]. The examples in this study may serve as an archetype in future research for documenting the conservation status and ensuring sustainability of all medicinal plant species across sub-Saharan African countries. Semenya et al. [87] documented 37 Indigenous anti-venereal plant species from 33 genera belonging to 24 families that are used by 34 Bapedi healers in 3 districts of Limpopo Province, South Africa, and highlighted several conservation strategies for the sustainable use of these medicinal plants. The authors reported that most of the plant species are not endangered nor threatened, while only five of the plant species recorded in the study appear on the South African National Red Data List as close to a threatened category. However, a study on Indigenous knowledge and techniques of herbalists related to the treatment of HIV/AIDS opportunistic infections in Uganda revealed that certain documented plant species are listed as threatened under the Nationally Threatened Species for Uganda [88]. The authors raised even much more concern about threat risk for the ones that have not been red-listed for lack of scientific assessment or due to deficient data on the medicinal plants.

Semenya et al. [87] identified the major factors threatening medicinal plant species used by Bapedi healers to be urban development, trading and agricultural expansion, deforestation and overexploitation. They concluded that the healers need to practice cultivation of important Indigenous plants in home gardens and be educated about the conservation measures they can implement to ensure the long-term sustainability of threatened and protected species, and ultimately traditional healing as a profession. Furthermore, it is observed that, even in the twenty-first century, Indigenous cultural norms such as the spiritually protected forests sustained through diverse taboos are still being revered and their prohibition laws complied to much better than government laws and policies. The comparatively less compliance to government laws and policies might be as a result of ignorance about the policies on the part of Indigenous communities and/or lack of concerted effort to enforce the regulations on the part of the government. Other cultural norms noted as viable conservation strategies are social structure management, night and naked restricted harvesting, i.e., having access to collect plants only at night and while naked, gender restricted harvesting, and nature-related spiritual knowledge [89–92]. Nevertheless, it is imperative that Indigenous cultural values be adapted into national biodiversity conservation agenda due to the exceptional role of culture in facilitating medicinal plant conservation [24, 89, 90].

#### Sustainability of medicinal plant resources

The root is the major plant part used for the treatment of venereal diseases (Fig. 4A), an indication that destructive harvesting may be indulged by plant harvesters/gatherers and traditional healers in African communities. Root, stem bark, and whole-plant harvesting is destructive to medicinal plants compared to collecting their leaves and flowers. Destructive harvesting ultimately leads to resource exhaustion and potential species extinction for medicinal plants with slow growth and low availability status, if left unchecked [24, 88, 93, 94]. However, van Andel et al. [95] noted that it is important to conduct field studies on abundances of species, and the effect of various extraction/collection methods on their survival, reproduction and growth to ascertain whether the commercial harvesting of seeds, roots or stem is a destructive activity. For instance, bark harvesting could be sustainable in as much as ring-barking is not practiced or trees not felled and the tree species can quickly recover. This is typical of the impressive bark recovery rates and post 24-month complete wound closing of previously untouched species of *Khaya senegalensis*. This species seems to be capable of supporting sustainable bark harvesting due to its rapid growth in open areas and resilience to debarking. On the other hand, van Andel et al. [95] reported that *Pterocarpus erinaceous* responds comparatively slowly to bark removal, but for the fact that the species can sprout again after felling, it is suggested that the trees be cut at 1 m height and their bark peeled, which would make them coppice from the trunks and generate new shoots over time. Therefore, there is a need for further research on the ethnobotanically valued anti-venereal plants (Table 2) to ascertain the ecological impact of their organ harvesting (parts used as ethnomedicine) and their adaptability to cultivation.

According to the National Red List Project (https://www.nationalredlist.org/), there are no reports of any African country with available directory that provides updated data on the conservation status of medicinal plants other than South Africa (National Red Data List), Angola (Lista Vermelha de especies de Angola: Extintas, ameaçadas de extinção, vulneráveis e invasoras), and Uganda (Nationally Threatened Species for Uganda). Although this suggests the need for other African countries to step up their conservation efforts, there are various effective strategies that can be adopted apart from the utilisation of cultivation and agroforestry practices, and community-based management [77]. For instance, assessments of plant species to determine the conservation status and prioritise conservation efforts can be conducted. Laws and regulations to protect threatened plant species and their habitats can also be implemented. Furthermore, the sustainable use of plant species can be promoted and awareness raised about the importance of their conservation. Collaborating with other countries to share information and coordinate conservation efforts is another pertinent strategy. A combination of approaches will be necessary to ascertain the sustainable use of valuable medicinal plant resources [95, 96]. The use of these strategies could help safeguard plant species in African countries and support conservation efforts at regional and international levels.

### Treatment of venereal diseases in African traditional medicine (ATM): adaptation and integration into modern medicine

#### Dosage and toxicity in African traditional medicine (ATM)

In this review, some studies documented the traditional posology of some anti-venereal herbal preparations [30, 34, 35, 39, 49] while others did not. Against popular belief about the lack of quantifiable administration in traditional medicine, this suggests that African traditional healers do have idea about dosage, i.e., posology is also applied in ATM although it is not clear how it is determined in the selected studies (Table 2). In fact, a body of ethnobotanical research, separate from this study, has demonstrated that traditional medicine practitioners (TMPs) do factor in dosage when prescribing treatments [97–100]. However, Ezekwesili-Ofili and Okaka [101] noted that TMPs determine herbal preparation dosages by evidence-based observation over time. Meanwhile, observation is a fundamental element of scientific research, providing scientists with basis to test hypotheses and theories. This reveals a similitude between ATM and modern medicine. For instance, in ancient times (in Africa, particularly among the Yorùbá people), the ingestibility of forest products (fruits, vegetables, mushrooms) was determined by first giving them to domestic animals and observing them for specified duration. The complications arising from these feeds in the animals was used to adjudge toxicity. Thus, incorporating traditional medicine practices into modern healthcare systems might not be out of place and could serve as a means to preserve some viable Indigenous knowledge techniques and enhance the management of VDs.

#### Standardization of herbal medicine

Several studies have demonstrated how traditional practices could be adapted or integrated into modern medical practices. Mokgobi [102] recommended that formal integration efforts be made through collaboration between Western hospitals and traditional healers in South Africa. Similarly, Maluleka and Ngoepe [103] emphasised a position of collaboration that encourages two-way referrals of patients between government hospitals and traditional healers. However, it is difficult not to indulge scepticism in the future success of this kind of collaboration between traditional physicians and Western doctors in Africa because of the divergence in the conceptualisation of illness that exists between the African traditional healing model and the Western healing model [102]. The perspective of aetiology, diagnosis and treatment of diseases between these systems differs to a large extent, and it can only be rationalised that both systems should be allowed to operate as distinct entities within a national framework that recognises traditional healing in mainstream health system as opined by Gbadamosi and Egunyomi [35]. Apart from this, the distrust between traditional healers and western medicine practitioners is a huge factor in this scepticism. Gbadamosi and Egunyomi [35] mentioned that traditional healers believe that western doctors undermine their work, and that western practitioners lack knowledge about traditional theories of disease and health, resulting in mistrust between both sides. A much more feasible way to integrate traditional medicine into modern medicine is through the documentation and validation/valorisation of traditional medicine practices, noting that research efforts on the standardisation of herbal medicine can help in its integration into modern medicine [104, 105]. Pirintsos et al. [106] reaffirmed that traditional ethnopharmacology has provided an early framework for the therapeutic use of natural compounds.

#### Indigenous knowledge techniques and drug discovery

Several studies have demonstrated how researchers have focused on drug discovery from herbal medicines or botanical sources [107–109]. These studies provided examples of successful drug discovery from botanical sources, such as the development of guggulsterones from *Commiphora mukul* Engl. for hyperlipidaemia,  and antimalarial drugs from *Artemisia alba* Turra and *Artemisia annua* L. However, Gertsch [110] argued that the late twentieth century uncritical passing of virtually all anecdotal evidence (ethnomedicine) as scientifically proven—by the over-interpreting of pharmacological data (particularly *in* *vitro* assays) leading to futile bioprospecting and bankruptcy of a certain pharmaceutical company in 2001—still hampers the veracity of ethnopharmacological research. The author opined that despite the exponential growth and interest in ethnopharmacology over the past few decades, medicinal plant research has had no significant contribution to drug discovery, unlike in the eighteenth and nineteenth centuries, due to several factors such as ambiguous claims of prospective therapeutic agents, which frequently lack objectivity and may be prone to false assertions; the desperation of ethnopharmacologists to prove their theories right, which makes them uncritical with their data; and careless execution of bioassays by engaging outrageously high concentrations of a plant extract to determine a pharmacological response [111].

To resolve this issue in the twenty-first century, the use of new technologies such as genomics, metabolomics/metabonomics, proteomics, transcriptomics, automation, and computational strategies will surely pave the way for inventive drug design leading to better drug candidates [108]. Gertsch [110] also recommended the need for interdisciplinary (industrial and academic) collaborations to garner a critical volume of sound data on the pharmacology and chemistry of a plant, facilitating the ascertainment of whether the plant compounds are suitable for the development of botanical drugs, the development of food additives or dietary supplement, or the development of cosmeceuticals. Drawing upon the insights of Tresina et al. [108] and Gertsch [110], it is evident that the bioprospecting potential of medicinal plants in sub-Saharan Africa is substantial. This potential is largely attributable to the region's rich biodiversity and the extensive traditional knowledge associated with these plants. Consequently, these factors present a significant opportunity for sustainable drug development. Furthermore, they could serve as a valuable resource for the discovery of novel drugs aimed at combating VDs. To ensure equitable benefits, strategies to prevent biopiracy are crucial. This includes establishing legal frameworks for access and benefit-sharing and promoting Indigenous participation in bioprospecting activities [112–115].

In the pursuit of incorporating traditional medicine into modern medicine, a step further from pharmacological screening would be the modernised packaging of traditional medicine, even though the quantity of administering medication differs from patient to patient [103]. In this way, the adaptation of traditional medicine practices into modern medicine might not only provide opportunities for the preservation and utilization of indigenous knowledge techniques among African communities but also help in the standardization of anti-venereal herbal medicine and substantiate means for drug leads and novel drugs.

### Research gap: ethnobotany and ethnopharmacology

#### Ethnobotanical studies

This review focused on 7 prevalent VDs in sub-Saharan Africa of which a significant number of ethnobotanical uses were found for gonorrhoea, syphilis, chlamydia, and HIV/AIDS. However, there were few published results available for genital warts and genital herpes. Notably, no records were found that met the eligibility criteria for the ethnobotanical uses of plants for trichomoniasis in any sub-Saharan African country. The reason for this lack of ethnobotanical information on *Trichomonas vaginalis* infection remains unclear. Nevertheless, a preliminary ethnopharmacological investigation from South Africa has been conducted on trichomoniasis. A few plant species, including *Bidens pilosa* L. leaves, *Sclerocarya birrea* Hochst bark, *Syzygium cordatum* Hochst bark, *Tabernaemontana elegans* Stapf. bark, and *Ozoroa engleri* R.Fern. & A. Fern. leaves showed some degree of bioactivity against the pathogen [116]. While many Africans depend on medicinal plants, ethnobotanical studies are often an underpinning for ethnopharmacological research [14, 15, 68, 101, 117]. Due to the limitation of this review study, there is a recognised need for more ethnobotanical surveys focusing on trichomoniasis, genital warts, and genital herpes. These surveys should include pertinent ethnobotanical indices and conservation information, and ensure their accessibility through academic publication.

#### Ethnopharmacological research

Over the past decade—2013 till date (May 16, 2024) when the ethnopharmacological review was conducted, 15 studies originating from sub-Saharan Africa have been found published on the pharmacological potential of anti-venereal plants [116, 118–131]. The juxtaposition of the 44 plant species investigated by these studies with the recorded plants in Table 2 showed that only 16 of the recorded plant species have been evaluated for their preliminary pharmacodynamic activities. And out of these 16 plants, 14 revealed significant anti-venereal potency (Table 3).

**Table 3:** Pharmacological potential of anti-venereal plants in sub-Saharan Africa published from 2013 to May 16, 2024

Botanical name	Common name	Country of study	Pharmacodynamic effect of plant materials	Venereal diseases treated	
				Pharmacological studies	Ethnobotanical studies
*Abrus precatorius L	Rosary pea	South Africa	The ethanolic extract of the aerial parts and seeds showed an MIC value of 6.3 mg/mL against *Neisseria gonorrhea* [128]	Gonorrhea	Genital herpes
*Aloe marlothii A.Berger	Flat-flowered aloe	South Africa	The organic and aqueous leaves extract showed MIC values of 6 and > 16 mg/mL, respectively against clinical strain of *Trichomonas vaginalis*; 1 and > 16 mg/mL, respectively, against *N*. *gonorrhoeae* [116]	Gonorrhea; Trichomoniasis	Gonorrhea; Chlamydia; Genital herpes
*Cassia abbreviata* Oliv	Long-tail cassia	South Africa	The aqueous bark extract exhibited < 40% HIV-RT enzyme inhibition at doses 50 and 100 µg/mL [127]. However, the ethanol and aqueous root extracts were active against *N. gonorrhoeae*, with MIC values of 125 and 62.5 µg/ml, respectively [130]	HIV, Gonorrhea	Gonorrhea; Syphilis; HIV/AIDS
*Agave sisalana* Perrine	Sisal	South Africa	The acetone and methanolic roots extract demonstrated notable antigonococcal activity with an MIC value of 1.30 mg/mL each [121]	Gonorrhea	Syphilis;
*Catharanthus roseus* (L.) G.Don	Madagascar periwinkle	South Africa	The methanolic and dichloromethane roots extract showed good inhibitory activity against *N. gonorrhoea* with MIC values of 0.63 and 1.3 mg/mL, respectively [121], whereas the acetone roots extract at a dose of 0.05 mg/mL demonstrated 95.5% inhibition of the pathogen [124]. The methanolic extract of the whole plant elicited significant anti-HSV-1 activity with EC50 values of 0.0138, 0.025 and 0.106 mg/mL in post-treatment, pre-treatment and virucidal assays [118]	Gonorrhea; Genital herpes	Gonorrhea; Genital warts
*Hypoxis hemerocallidea* Fisch., C.A.Mey. & Avé-Lall	African potato	South Africa	The aqueous extract of the corm exhibited notable antigonococcal activity with an MIC value of 0.50 mg/mL [116]	Gonorrhea	HIV/AIDS
*Kigelia africana* (Lam.) Benth	Sausage tree	South Africa	The ethyl acetate, dichloromethane and methanolic leaf extracts elicited considerable inhibitory activity against HIV-1 RT with inhibition percentages of 65%, 70% and 60% respectively, at a dose of 100 µg/mL [126]	HIV	Syphilis; Genital herpes
*Opuntia ficus-indica* (L.) Mill	Prickly pear	South Africa	The dichloromethane and methanolic roots extract demonstrated notable antigonococcal activity with MIC values of 0.97 and 1.3 mg/mL, respectively [121]	Gonorrhea	Gonorrhea
*Peltophorum africanum* Sond	African wattle	South Africa	The aqueous and the organic root extracts showed remarkable MIC values of 0.50 and 0.25 µg/mL, respectively, against *N. gonorrhoeae* [116]	Gonorrhea	Gonorrhoea, Syphilis
*Pentanisia prunelloides* (Klotzsch) Walp	Wild verbena or broad-leaved Pentanisia	South Africa	The ethanolic root extract showed an MIC value of 0.78 mg/mL against *N. gonorrhoeae* [131]	Gonorrhea	Gonorrhea
*Ricinus communis* L	Castor bean	South Africa	The dichloromethane and methanolic roots extract elicited significant inhibitory activity against *N*. *gonorrhea* with MIC values of 1.3 and 0.97 mg/mL, respectively [121]	Gonorrhea	Gonorrhea; Chlamydia
*Senna didymobotrya* (Fresen.) H.S.Irwin & Barneby	Popcorn cassia	South Africa	The acetone and methanolic roots extract exhibited considerable antigonococcal activity with an MIC value of 1.30 mg/mL each [121]	Gonorrhea	Chlamydia
*Solanum elaeagnifolium* Cav	Silverleaf nightshade	South Africa	The dichloromethane and methanolic roots extract showed considerable inhibitory activity against *N*. *gonorrhoeae* with an MIC value of 1.3 mg/mL each [121]	Gonorrhea	Syphilis
*Solanum mauritianum* Scop	Bugweed	South Africa	The dichloromethane roots extract demonstrated notable inhibitory activity against *N. gonorrhea* with an MIC value of 1.3 mg/mL [121]	Gonorrhea	Syphilis; HIV/AIDS
*Terminalia sericea* Burch. ex DC	Silver cluster-leaf	South Africa	The 70% ethanolic root extract, at a concentration of 102.8 μg/mL, showed 100% HIV-1 RT inhibition activity compared to the positive control doxorubicin, 96.5% [122]. The ethyl acetate, dichloromethane and methanolic leaves extracts also showed considerable inhibitory activity (> 70%) against HIV-1 RT at a dose of 100 µg/mL. And on the other hand, the methanolic leaves extract demonstrated good inhibitory activity against *N. gonorrhoeae* with an MIC value of 0.8 mg/mL. [126]	Gonorrhea; HIV	Syphilis
*Ximenia caffra* Sond	Sour plum	South Africa	The organic extract of the leaves exhibited notable antigonococcal activity with an MIC value of 0.63 mg/mL [116]. The dichloromethane and n-hexane fractions of the ethanolic leaves extract demonstrated 78.8% and 73.4% inhibition, respectively, against *N. gonorrhoeae* [129]	Gonorrhea	Gonorrhea; Syphilis

^*^asterisked plants are the plants with no significant pharmacodynamic (anti-venereal) potential

Although the bark and root of *Cassia abbreviata* Oliv. are used ethnobotanically for gonorrhoea, syphilis, chlamydia, and HIV/AIDS (Table 2), the root had low inhibitory activity against HIV-RT enzyme [127]. It is yet to be verified if the active compounds in the bark are the one responsible for the ethnobotanical anti-HIV potency or if the root could still have good inhibitory activity against other HIV enzymes such as HIV-PR, HIV-IN and HIV-RNase H. Therefore, there arises a need for extensive and explorative ethnopharmacological research on all the recorded plants from the ethnobotanical surveys (Table 2).

## Conclusion

In this review, the richness of Indigenous knowledge and high diversity of medicinal plant species, majorly prepared by decoction and infusion, for treating gonorrhoea, HIV/AIDS, and syphilis was recorded. However, there was sparsely available ethnobotanical information on genital warts, genital herpes, and chlamydia. The review revealed a need for ethnobotanical surveys on trichomoniasis in sub-Saharan Africa. These findings are important for preserving Indigenous knowledge before it disappears due to lack of documentation and for identifying anti-venereal plants for further ethnopharmacological evaluation. Conservation issues arising from typically unsustainable harvesting due to the major plant parts used—roots—in this review were also unveiled. Although the number of ethnobotanical studies reviewed in this paper might seem small at first glance—especially considering the vast geographical region of sub-Saharan Africa—the paper focused specifically on VDs, which have been less commonly explored in ethnobotanical research. In addition, the 20 reviewed studies represent the most relevant and available studies on the topic and provide a robust dataset for understanding the use of medicinal plants for VD management in sub-Saharan Africa. Furthermore, the diversity and richness of the plants (445 species) from over 872 Indigenous knowledge holders across 10 countries suggest that our sample size is sufficient to draw meaningful conclusions. Regulations for the protection of threatened plant species as well as collaboration among countries in sub-Saharan Africa for information sharing on conservation efforts and the coordination of ethnobotanical research cannot be over-emphasised. Interdisciplinary collaborations necessary for curating a critical volume of quality data on the pharmacology and chemistry of a plant, which could determine the exact pharmaceutical suitability of the plant compounds are also recommended. Finally, this review provided evidence to support the hypotheses that Indigenous cultures have a rich tradition of using Indigenous knowledge and plants for the treatment of VDs and that these practices can be adapted and integrated into modern medical practices to provide effective and culturally sensitive treatment options.

## Supplementary Information

Supplementary material 1.

## Data Availability

Additional data have been included in the supplementary file.
