# Supplementary material for: Plants used for the management of venereal diseases in sub-Saharan Africa: a systematic review and critical assessment of their research status
Source: Trop Med Health. 2024 Dec 26;52:100. doi: 10.1186/s41182-024-00651-y (PMC11670375; doi:10.1186/s41182-024-00651-y)
Supplement: Supplementary file 1 — Supplementary material 1. [file 41182_2024_651_MOESM1_ESM.docx]

**Supplementary Table S1:** A compilation of 445 medicinal plant species used for treating specific venereal diseases across sub-Saharan Africa. All botanical names were verified with the World Flora Online ([www.worldfloraonline.org](http://www.worldfloraonline.org/)), and any unindexed plant was further verified with the Medicinal Plant Names Services portal (<http://mpns.kew.org/mpns-portal>).

| **Botanical names** | **Families** | **Plant part used** | **Country of study: selection criteria (SC) and ethnobotanical studies (references)** | **SC index** | **Venereal diseases treated** |
| --- | --- | --- | --- | --- | --- |
| *Abrus precatorius* L. | Fabaceae | Root; whole plant | Tanzania: Ci [1]; Zambia: Fi_1_ [2]; Fi_2_ [3]; RFC [4] | Ci: 34.8;  Fi_1_: 20; Fi_2_:  60; RFC:  0.2 | Gonorrhea (Ci, Fi_1_, Fi_2_, RFC); Chlamydia (Fi_1_); Syphilis (Fi_1_) (Fi_2_); Genital warts (Fi_1_); Genital herpes (Fi_2_) |
| *Acacia mearnsii* De Willd. | Fabaceae | Stem bark | Uganda: Fm [5] | 1 | HIV/AIDS |
| *Acalypha villicaulis* Hochst. ex A.Rich. | Euphorbiaceae | Whole root | Uganda: Fm [5] | 3 | HIV/AIDS |
| *Acanthus montanus* (Nees) T.Anders | Acanthaceae | Leaves | Cameroon: Fi [6] | 6 | HIV/AIDS |
| *Achyranthes aspera* L. | Amaranthaceae | Root; fruit and seed | Zambia: Fi [3]; Ethiopia: Fc [7] | Fi: 20; Fc:  1 | Genital warts (Fi); Gonorrhoea (Fc) |
| *Acokanthera schimperi* (A.DC.) Benth. & Hook.f. ex Schweinf. | Apocynaceae | Leaves, root, root bark, stem bark, and seed | Ethiopia: Fc [7] | 5 | Gonorrhoea |
| *Adansonia digitata* L. | Malvaceae | Stem bark | Tanzania: Ci [1] | 13 | HIV/AIDS |
| *Adenia cissampeloides* (Planch. ex Benth.) Harms | Passifloraceae | Root bark; stem bark; leaves | Uganda: Fm [5] | 1 | HIV/AIDS |
| *Aerva javanica* Juss. | Amaranthaceae | Root | Ethiopia: Fc [7] | 1 | Gonorrhoea |
| *Agave americana* L. | Asparagaceae | Leaves | South Africa: FL [8] | 33.3 | Gonorrhoea; Chlamydia; Syphilis |

| **Botanical names** | **Families** | **Plant part used** | **Country of study: selection criteria (SC) and ethnobotanical studies (references)** | **SC index** | **Venereal diseases treated** |
| --- | --- | --- | --- | --- | --- |
| *Agave sisalana* Perrine | Asparagaceae | Root | South Africa: FL_1_, FL_2_, FL_3_, FL_4_ [8]; Fu [9] | FL_1_, FL_2_:  33.3; FL_3_:  16.7; FL_4_:  8.3; Fu: 11 | Gonorrhoea (FL_1_, Fu); Chlamydia (FL_2_); Syphilis (FL_3_); Genital warts (FL_4_) |
| *Ageratum conyzoides* L. | Asteraceae | Root | Ethiopia: Fc [7]; Nigeria: Fu [10]; Uganda: Fm [5]; Cameroon: Fi [6] | Fc: 1; Fi:  4.6; Fm: 1;  Fu: 5 | Gonorrhoea (Fc, Fu); HIV/AIDS (Fi, Fm) |
| *Ageratum houstonianum* Mill. | Asteraceae | Root | South Africa: Fc [9] | 3 | Gonorrhoea |
| *Albizia amara* (Roxb.) Boivin | Fabaceae | Stem bark | Zambia: Fi [3] | 60 | Gonorrhoea |
| *Albizia anthelmintica* (A. Rich.) Brogn. | Fabaceae | Root | Ethiopia: Fc [7] | 1 | Gonorrhoea |
| *Albizia coriaria* Welw. ex Oliv. | Fabaceae | Stem bark | Uganda: Fm [5] | 3 | HIV/AIDS |
| *Albizia versicolor* Welw. ex Oliv. | Fabaceae | Stem bark | Zambia: Fi [2] | 15 | Gonorrhoea; Chlamydia |
| *Alchornea cordifolia*  (Schumach.) Müll.Arg. | Euphorbiaceae | Leaves | Nigeria: Fc [11] | 38 | Gonorrhoea |
| *Allanblackia floribunda* Oliv. | Clusiaceae | Stem bark | Cameroon: Fi [6] | 2 | HIV/AIDS |
| *Allium sativum* L. | Amaryllidaceae | Bulb | Ethiopia: Fc [7]; Cameroon: Fi [6] | Fc: 1; Fi:  5.3 | Gonorrhoea (Fc); HIV/AIDS (Fi) |
| *Allium schoenoprasum* L. | Amaryllidaceae | Leaves | Nigeria: Fc [11] | 15 | Gonorrhoea |
| *Aloe arborescens* Mill. | Asphodelaceae | Root | South Africa: Ci [12] | 3 | HIV/AIDS |
| *Aloe falcata* Baker | Asphodelaceae | Root | South Africa: Ci [12] | 3 | HIV/AIDS |

| **Botanical names** | **Families** | **Plant part used** | **Country of study: selection criteria (SC) and ethnobotanical studies (references)** | **SC index** | **Venereal diseases treated** |
| --- | --- | --- | --- | --- | --- |
| *Aloe marlothii* A.Berger | Asphodelaceae | Root; leaves | South Africa: Ci [12]; Botswana: Fi [13] | Ci: 24; Fi:  N/A | Gonorrhoea (Ci, Fi); Chlamydia (Ci) |
| *Aloe marlothii* subsp. *marlothii* | Asphodelaceae | Root; leaves | South Africa: Fi [14] | Fi: 13.3 | Gonorrhoea |
| *Aloe schweinfurthii* Baker Synonym: *Aloe barteri* Backer | Asphodelaceae | Leaves | Cameroon: Fi [6] | 13.2 | HIV/AIDS |
| *Aloe secundiflora* Engl. | Asphodelaceae | Leaves | Kenya: ICf [15] | 0.6 | HIV/AIDS |
| *Aloe* sp*.* | Asphodelaceae | Leaves and root bark | Uganda: Fm [5] | 7 | HIV/AIDS |
| *Aloe trichosantha* A.Berger | Asphodelaceae | Latex | Ethiopia: Fc [7] | 1 | Gonorrhoea |
| *Aloe zebrina* Baker | Asphodelaceae | Root | Botswana: Fi [13] | N/A | HIV/AIDS |
| *Alstonia boonei* De Wild. | Apocynaceae | Stem bark | Uganda: Fm [5]; Nigeria: Fu [10] | Fm, Fu: 1 | Gonorrhoea; HIV/AIDS |
| *Alternanthera pungens* Kunth | Amaranthaceae | Tuber | South Africa: Fi [14]; Ci [12] | Fi: 3.3; Ci:  3 | Gonorrhoea (Fi); Chlamydia (Ci) |
| *Ampelocissus obtusata* Planch. | Vitaceae | Root | Zambia: Fi [2] | 25 | Syphilis; Genital herpes |
| *Anacardium occidentale* L. | Anacardiaceae | Leaves; stem bark | Nigeria: Fc [11], UMi [16] | Fc: 95;  UMi: 26.3 | Gonorrhoea (Fc); Syphilis (UMi) |
| *Ananas comosus* (L.) Merr.; Synonym: *Ananas sativa* Lindl. | Bromeliaceae | Fruit | Nigeria: Fu [10]; Uganda: Fm [5] | Fm: 1; Fu:  2 | Gonorrhoea (Fu); HIV/AIDS (Fm) |
| *Anonychium africanum* (Guill*.* &amp; Perr.) C.E.Hughes &amp; G.P.Lewis; Synonym: | Fabaceae | Root | Nigeria: Fi [17] | 1 | Gonorrhoea |

| **Botanical names** | **Families** | **Plant part used** | **Country of study: selection criteria (SC) and ethnobotanical studies (references)** | **SC index** | **Venereal diseases treated** |
| --- | --- | --- | --- | --- | --- |
| *Prosopis africana* (Guill &amp; Perr) Taub. |  |  |  |  |  |
| *Anredera cordifolia* (Ten.) Steenis | Basellaceae | Aerial tuber | South Africa: FL_1_, FL_2_, FL_3_, FL_3_, FL_5_  [9] | FL_1_: 33.3; FL_2_, FL_3_, FL_4_, FL_5_: 16.7 | Gonorrhoea (FL_1_); Chlamydia (FL_2_) Syphilis (FL_3_); HIV/AIDS (FL_4_);  Genital warts (FL_5_) |
| *Anthocleista djalonensis*  A.Chev. | Gentianaceae | Root | Nigeria: Fc [11]; UMi [16] | Fc: 79;  UMi: 31.3 | Gonorrhoea |
| *Argemone ochroleuca* Sweet | Papaveraceae | Leaves; root; whole plant | South Africa: Fu [9] | 11 | Gonorrhoea |
| *Aristolochia bracteolata* Lam. | Aristolochiaceae | Root | Nigeria: Fu [10] | 4 | Gonorrhoea |
| *Aristolochia repens* Mill. | Aristolochiaceae | Root | Nigeria: UMi [16] | 37.5 | Gonorrhoea |
| *Artemisia afra* Jacq. ex Willd. | Asteraceae | Stem and leaves | Ethiopia: Fc [7] | 1 | Gonorrhoea |
| *Artemisia annua* L. | Asteraceae | Leaves | Cameroon: Fi [6] | 13.8 | HIV/AIDS |
| *Asepalum eriantherum* (Vatke) Marais | Orobanchaceae | Root | Ethiopia: Fc [7] | 1 | Gonorrhoea |
| *Asparagus falcatus* L. | Asparagaceae | Root | Tanzania: Ci [1] | 26.1 | Gonorrhoea |
| *Asparagus microraphis* Baker | Asparagaceae | Root | South Africa: UV [18] | 0.04 | HIV/AIDS |
| *Asparagus racemosus* Willd. | Asparagaceae | Whole plant | Zambia: Fi [3] | 20 | Syphilis |

| **Botanical names** | **Families** | **Plant part used** | **Country of study: selection criteria (SC) and ethnobotanical studies (references)** | **SC index** | **Venereal diseases treated** |
| --- | --- | --- | --- | --- | --- |
| *Aspilia africana* (Pers.) C.D.Adams | Asteraceae | Leaves; root | Cameroon: Fi [6] | 5.3 | HIV/AIDS |
| *Aspilia kotschyi* (Sch.Bip. ex Hochst.) Oliv. | Asteraceae | Leaves | Uganda: Fm [5] | 1 | HIV/AIDS |
| *Asystasia vogeliana* Benth. | Acanthaceae | Leaves | Cameroon: Fi [6] | 6.6 | HIV/AIDS |
| *Azadirachta indica* A.Juss. | Meliaceae | Leaves; whole plant | Ethiopia: Fc [7]; Uganda: Fm [5] | Fc: 1; Fm:  3 | Gonorrhoea (Fc); HIV/AIDS (Fm) |
| *Baccharoides adoensis* (Sch.Bip. ex Walp.) H.Rob. Synonym: *Vernonia adoensis* Sch.Bip. ex Walp. | Asteraceae | Leaves and root | Nigeria: Fu [10] | 4 | Gonorrhoea |
| *Baccharoides calvoana* var. *hymenolepis* (A.Rich.) Isawumi Synonym: *Vernonia hymenolepis* A.Rich | Asteraceae | Leaves | Ethiopia: Fc [7] | 1 | Gonorrhoea |
| *Baccharoides guineensis* (Benth.) H.Rob. Synonym: *Vernonia guineensis* Benth. | Asteraceae | Leaves; tubers | Cameroon: Fi [6] | 9.3 | HIV/AIDS |
| *Baikiaea plurijuga* Harms | Fabaceae | Stem bark | Zambia: Fi [2] | 30 | Syphilis |
| *Baphia nitida* G.Lodd. | Fabaceae | Leaves | Nigeria: Fc [11] | 32 | Gonorrhoea |
| *Barleria spinulosa* subsp. kirkii (T.Anderson) I.Darbysh.  Synonym: *Barleria kirkii* T. Anderson | Acanthaceae | Leaves | Zambia: Fi [3] | 13.3 | HIV/AIDS |

| **Botanical names** | **Families** | **Plant part used** | **Country of study: selection criteria (SC) and ethnobotanical studies (references)** | **SC index** | **Venereal diseases treated** |
| --- | --- | --- | --- | --- | --- |
| *Bartsia* sp*.* | Orobanchaceae | Leaves | Uganda: Fm [5] | 1 | HIV/AIDS |
| *Berkheya montana* J.M.Wood &amp; M.S.Evans | Asteraceae | Root | South Africa: UV [18] | 0.04 | Gonorrhoea |
| *Beta vulgaris* L. | Amaranthaceae | Root | Cameroon: Fi [6] | 4 | HIV/AIDS |
| *Biancaea decapetala* (Roth) O.Deg. Synonym: *Caesalpinia decapetala* (Roth)  Alston | Fabaceae | Root | South Africa: Fi [14], Ci [12] | Fi: 3.3; Ci:  3 | Gonorrhoea |
| *Bidens pilosa* L. | Asteraceae | Root; leaves | Ethiopia: Fc [7]; Cameroon: Fi [6]; Uganda: Fm [5] | Fc:1; Fi: 4; Fm:2 | Gonorrhoea (Fc); HIV/AIDS (Fi, Fm) |
| *Bidens* sp.* | Asteraceae | Whole root | Uganda: Fm [5] | 1 | HIV/AIDS |
| *Boerhavia sinuata* (Meikle) Greuter & Burdet Synonym: *Commicarpus sinuatus* Meikle | Nyctaginaceae | Leaves and fruit | Ethiopia: Fc [7] | 1 | Gonorrhoea |
| *Bombax buonopozense*  P.Beauv. | Malvaceae | Stem bark | Cameroon: Fi [6] | 2.6 | HIV/AIDS |
| *Boscia albitrunca* Burch. Gilg&Benedict | Capparaceae | Root; leaves and stem bark | Zambia: Fi_1_ [2]; RFC [4]; South Africa: Ci [12]; Namibia: Fi_2_ [19] | Fi_1_: 50; Fi_2_:  89; Ci: 3;  RFC: 0.4 | Syphilis (Fi_1_, Fi_2_, RFC); HIV/AIDS (Ci, Fi, RFC) |
| *Boscia salicifolia* Oliv. | Capparaceae | Root | Zambia: Fi [3] | 70 | Syphilis; HIV/AIDS |
| *Boswellia dalzielii* Hutch. | Burseraceae | Leaves and stem bark | Nigeria: Fi [20] | 13 | HIV/AIDS |
| *Brassica oleracea* L. | Brassicaceae | Leaves | Cameroon: Fi [6] | 2 | HIV/AIDS |

| **Botanical names** | **Families** | **Plant part used** | **Country of study: selection criteria (SC) and ethnobotanical studies (references)** | **SC index** | **Venereal diseases treated** |
| --- | --- | --- | --- | --- | --- |
| *Bridelia cathartica* Bertol. | Phyllanthaceae | Leaves and fruit; stem bark | Zambia: Fi [3] | 13.3 | Gonorrhoea |
| *Bridelia micrantha* (Hochst.) Baill. | Euphorbiaceae | Stem bark; whole root | Kenya: ICf [15]; Uganda: Fm [5] | ICf: 0.6;  Fm: 1 | HIV/AIDS |
| *Brucea antidysenterica*  J.F.Mill. | Simaroubaceae | Leaves and seed | Ethiopia: Fc [7] | 3 | Gonorrhoea |
| *Bulbine narcissifolia* Salm- Dyck | Asphodelaceae | Leaves | South Africa: UV [18] | 0.29 | Gonorrhoea |
| *Burkea africana* Hook. | Fabaceae | Root; stem bark | Zambia: Fi [2]; South Africa: Ci [12] | Fi: 40; Ci:  3 | Gonorrhoea (Fi); HIV/AIDS (Ci, Fi) |
| *Callilepis salicifolia* Oliv. | Asteraceae | Tuber | South Africa: Fi [14]; Ci [12] | Fi: 3.3; Ci:  6 | Gonorrhoea (Ci, Fi); HIV/AIDS (Ci) |
| *Calpurnia aurea* (Aiton) Benth. | Fabaceae | Leaves and seed | Ethiopia: Fc [7] | 2 | Gonorrhoea |
| *Capparis erythrocarpos* Isert | Capparaceae | Whole root; stem bark | Uganda: Fm [5] | 2 | HIV/AIDS |
| *Capparis tomentosa* Lam. | Capparaceae | Root; root bark | Zambia: Fi_1_ [2]; Fi_2_ [3]; Uganda: Fm [5] | Fi_1_: 25; Fi_2_:  20; Fm: 2 | Syphilis (Fi_1_, Fi_2_); HIV/AIDS (Fi_1_, Fi_2_,  Fm); Genital herpes (Fi_2_) |
| *Caralluma peckii* P.R.O.Bally | Asclepiadaceae | Root | Ethiopia: Fc [7] | 1 | Gonorrhoea |
| *Carica papaya* L. | Caricaceae | Leaves; root | Uganda: Fm [5]; South Africa: Ci_1_ [12]; Fi [14]; Tanzania: Ci_2_ [1]; Nigeria: Fc [11] | Ci_1_: 3; Ci_2_:  8.7; Fm: 1;  Fi: 3.3; Fc:  10 | Gonorrhoea (Ci_1_, Ci_2_, Fi); Syphilis (Fc); HIV/AIDS (Fm) |

| **Botanical names** | **Families** | **Plant part used** | **Country of study: selection criteria (SC) and ethnobotanical studies (references)** | **SC index** | **Venereal diseases treated** |
| --- | --- | --- | --- | --- | --- |
| *Carissa spinarum* L.; Synonym: *Carissa edulis* Vahl | Apocynaceae | Root; root bark; stem bark | Ethiopia: Fc [7]; Uganda: Fm [5]; Nigeria: Fi [20] | Fc: 4; Fm:  3; Fi: N/A | Gonorrhoea (Fc); HIV/AIDS (Fm, N/A) |
| *Cassia abbreviata* Oliv. | Fabaceae | Root and stem bark | Zambia: Fi_1_ [2], Fi_2_ [3]; Tanzania: Ci [1] | Fi_1_: 60; Fi_2_:  83.3; Ci:  47.8 | Gonorrhoea (Ci, Fi_1_, Fi_2_); HIV/AIDS (Fi_1_);  Syphilis (Ci); Chlamydia (Ci) |
| *Cassia fistula* L. | Fabaceae | Leaves | Nigeria: UMi [16] | 18.8 | Gonorrhoea; Trichomoniasis |
| *Casuarina cunninghamiana*  Miq. | Casuarinaceae | Fruit | South Africa: Fu [9] | 3 | Gonorrhoea |
| *Catha edulis* (Vahl) Forssk. ex Endl. | Celastraceae | Stem and leaves | Ethiopia: Fc [7] | 4 | Gonorrhoea |
| *Catharanthus roseus* (L.) G.Don | Apocynaceae | Root; leaves | South Africa: Ci [12]; Fi [14]; Fu [9]; FL_1_, FL_2_, FL_3_, FL_4_, FL_5_ (Maema et al.,  2019) | Fi: 60; Ci:  82; Fu: 6;  FL_1_: 57.9;  FL_2_: 15.8; FL_3_, FL_4_: 10.5; FL5:  5.3 | Gonorrhoea (Ci, Fu, FL_1_); Syphilis (FL_2_); Chlamydia (FL_3_); Genital warts (FL_4_); HIV/AIDS (FL_5_) |
| *Catunaregam spinosa* (Thunb.) Tirveng. | Rubiaceae | Root | Tanzania: Ci [1] | 13 | Syphilis |
| *Celosia trigyna* L. | Amaranthaceae | Leaves | Ethiopia: Fc [7] | 1 | Gonorrhoea |
| *Centella asiatica* (L.) Urb. | Apiaceae | Leaves | Uganda: Fm [5] | 1 | HIV/AIDS |
| *Chamaecrista nigricans* (Vahl) Greene; Synonym: *Cassia*  *nigricans* Vahl | Caesalpiniaceae | Leaves | Uganda: Fm [5] | 1 | HIV/AIDS |

| **Botanical names** | **Families** | **Plant part used** | **Country of study: selection criteria (SC) and ethnobotanical studies (references)** | **SC index** | **Venereal diseases treated** |
| --- | --- | --- | --- | --- | --- |
| *Chenopodium opulifolium*  Schrad. ex W.D.J.Koch & Ziz | Amaranthaceae | Leaves | Uganda: Fm [5] | 1 | HIV/AIDS |
| *Chrysopogon nigritanus* (Benth.) Veldkamp; Synonym: *Vetiveria nigritana* (Benth.) Stapf | Poaceae | Root | Namibia: Fi [19] | 4 | Gonorrhoea |
| *Cinnamomum verum* J.Presl | Lauraceae | Root | South Africa: Ci [12] | 3 | HIV/AIDS |
| *Cissampelos mucronata*  A.Rich. | Menispermaceae | Root; leaves; whole plant | Zambia: Fi_1_ [2]; Fi_2_ [3] | Fi_1_: 35; Fi_2_:  73.3 | Syphilis |
| *Cissus quadrangularis* L. | Vitaceae | Root; sap; whole plant | Nigeria: Fc_1_, Fc_2_ [11]; Ethiopia: Fc_3_ [7]; Zambia: Fi [3] | Fi: 40; Fc_1_:  22; Fc_2_: 21;  Fc_3_: 1 | Gonorrhoea (Fi, Fc_1_, Fc_3_); Syphilis (Fc_2_); |
| *Cissus rotundifolia* Vahl | Vitaceae | Leaves | Ethiopia: Fc [7] | 1 | Gonorrhoea |
| *Citrullus colocynthis* (L.) Schrad. | Cucurbitaceae | Fruit | Nigeria: Fc_1_, Fc_2_ [11]; UMi [16] | Fc_1_: 56;  Fc_2_: 40;  UMi: 75 | Gonorrhoea (Fc_1_, UMi); Syphilis (Fc_2_, UMi) |
| *Citrullus lanatus* (Thunb.) Matsum. & Nakai | Cucurbitaceae | Root; fruit | South Africa: Ci [12]; Nigeria: Fu [10] | Ci: 3; Fu: 5 | HIV/AIDS (Ci);  Gonorrhoea (Fu); Syphilis (Fu) |
| *Citrullus naudinianus* Hook.f.; Synonym: *Acanthosicyos naudinianus* (Sond.) Jeffrey | Cucurbitaceae | Fruit | Namibia: Fi [19] | 71 | Gonorrhoea |
| *Citrus aurantifolia* (Christm.) Swingle | Rutaceae | Fruit | Nigeria: UMi [16]; Cameroon: Fi [6] | UMi: 50;  Fi: 9.8 | Gonorrhoea (UMi); HIV/AIDS (Fi) |

| **Botanical names** | **Families** | **Plant part used** | **Country of study: selection criteria (SC) and ethnobotanical studies (references)** | **SC index** | **Venereal diseases treated** |
| --- | --- | --- | --- | --- | --- |
| *Citrus limon* (L.) Osbeck; Synonym: *Citrus limonum* Risso | Rutaceae | Leaves; fruit | Uganda: Fm [5]; Cameroon: Fi [6] | Fm: 2; Fi:  4.6 | HIV/AIDS |
| *Clerodendrum capitatum*  (Willd.) Schumach. | Lamiaceae | Leaves and root | Zambia: Fi [3] | 60 | Gonorrhoea |
| *Clerodendrum umbellatum*  Poir. | Lamiaceae | Root | Ethiopia: Fc [7] | 1 | Gonorrhoea |
| *Cnestis ferruginea* (Vahl) DC. | Connaraceae | Root | Nigeria: UMi [16] | 25 | Gonorrhoea |
| *Coccinia abyssinica* (Lam.) Cogn. | Cucurbitaceae | Root | Ethiopia: Fc [7] | 1 | Gonorrhoea |
| *Cochlospermum planchonii*  Hook.f. ex Planch. | Bixaceae | Root | Nigeria: Fi [20] | N/A | HIV/AIDS |
| *Coffea arabica* L. | Rubiaceae | Leaves | Uganda: Fm [5] | 1 | HIV/AIDS |
| *Colophospermum mopane*  (Benth.) Leonard | Fabaceae | Stem bark | Zambia: Fi [2] | 15 | Syphilis |
| *Combretum hereroense* Schinz | Combretaceae | Leaves; root | Zambia: Fi1 [2]; Fi2 [3]; RFC [4] | Fi1: 60; Fi2:  53.3; RFC:  0.6 | Gonorrhoea (Fi1, Fi2, RFC); Chlamydia (Fi_2_) |
| *Combretum imberbe* Wawra | Combretaceae | Stem bark | Namibia: Fi [19] | 89 | Gonorrhoea |
| *Combretum molle* R.Br. ex G.Don | Combretaceae | Stem bark; root | Tanzania: Ci [1]; Uganda: Fm [5] | Fm: 1; Ci:  35 | Gonorrhoea (Ci); HIV/AIDS (Fm) |

| **Botanical names** | **Families** | **Plant part used** | **Country of study: selection criteria (SC) and ethnobotanical studies (references)** | **SC index** | **Venereal diseases treated** |
| --- | --- | --- | --- | --- | --- |
| *Combretum mossambicense*  Engl. | Combretaceae | Leaves; whole plant | Zambia: Fi_1_ (Chinsembu, 2016a); Fi_2_ [3] | Fi_1_: 35; Fi_2_:  36.7 | Gonorrhoea (Fi_1_, Fi_2_); Syphilis (Fi_1_, Fi_2_) |
| *Commelina benghalensis* L. | Commelinaceae | Whole plant | Uganda: Fm [5] | 1 | HIV/AIDS |
| *Commiphora angolensis* Engl | Burseraceae | Stem bark | Zambia: Fi [2] | 30 | Gonorrhoea; Chlamydia |
| *Corchorus tridens* L. | Malvaceae | Root; leaves and stem | Zambia: Fi_1_ [3]; Namibia: Fi_2_ [19] | Fi_1_: 13.3;  Fi_2_: 96 | Syphilis |
| *Cotyledon orbiculata* L. | Crassulaceae | Root | South Africa: Fi [14]; Ci [12] | Fi: 6.7; Ci:  3 | Gonorrhoea |
| *Crabbea velutina* S.Moore | Acanthaceae | Root | Ethiopia: Fc [7] | 1 | Gonorrhoea |
| *Crassocephalum crepidioides*  S.Moore | Asteraceae | Leaves | Nigeria: Fc [11]; Cameroon: Fi [6]; Uganda: Fm [5] | Fc: 34; Fi:  2; Fm: 1 | Gonorrhoea (Fc); HIV/AIDS (Fi, Fm) |
| *Crateva adansonii* DC. | Capparaceae | Root | Ethiopia: Fc [7] | 2 | Gonorrhoea |
| *Crotalaria glauca* Willd. | Papilionaceae | Root bark | Uganda: Fm [5] | 1 | HIV/AIDS |
| *Croton gratissimus* Burch. | Euphorbiaceae | Leaves; stem bark | Zambia: Fi_1_ [2]; Fi_2_ [3] | Fi_1_: 20; Fi_2_: 66.7 | Syphilis |
| *Croton macrostachyus* Hochst. ex Delile | Euphorbiaceae | Leaves and stem; stem bark; root; stem | Ethiopia: Fc [7] | 9 | Gonorrhoea |
| *Croton megalobotrys*  Müll.Arg. | Euphorbiaceae | Leaves | Zambia: Fi [3] | 60 | Gonorrhoea |
| *Cucumis dipsaceus* Ehrnb. ex Spach. | Cucurbitaceae | Leaves and root | Ethiopia: Fc [7] | 3 | Gonorrhoea |

| **Botanical names** | **Families** | **Plant part used** | **Country of study: selection criteria (SC) and ethnobotanical studies (references)** | **SC index** | **Venereal diseases treated** |
| --- | --- | --- | --- | --- | --- |
| *Cucumis ficifolius* A.Rich. | Cucurbitaceae | Root and fruit | Ethiopia: Fc [7] | 1 | Gonorrhoea |
| *Cucumis myriocarpus* Naudin; Synonym: *Cucumis myriocarpus* subsp. myriocarpus | Cucurbitaceae | Tuber | South Africa: Fi [14] | 3.3 | Gonorrhoea |
| *Cucumis myriocarpus* subsp. *leptodermis* (Schweick.) C.Jeffrey & P.Halliday | Cucurbitaceae | Tuber | South Africa: Ci [12] | 6 | Syphilis; Gonorrhoea |
| *Cucumis prophetarum* L. | Cucurbitaceae | Fruit | Ethiopia: Fc [7] | 1 | Gonorrhoea |
| *Cucurbita maxima* Lam. | Cucurbitaceae | Leaves | Cameroon: Fi [6] | 2.6 | HIV/AIDS |
| *Cucurbita pepo* L. | Cucurbitaceae | Leaves; seeds | Ethiopia: Fc [7]; Fi [6] | Fc: 1; Fi:  7.3 | Gonorrhoea (Fc); HIV/AIDS (Fi) |
| *Curculigo pilosa* (Schumach. & Thonn.) Engl. | Hypoxidaceae | Rhizome | Nigeria: Fu [10]; UMi [16] | Fu: 3;  UMi: 52.5 | Gonorrhoea |
| *Cussonia paniculata* Eckl. &amp; Zeyh. | Araliaceae | Root | South Africa: UV [18] | 0.08 | HIV/AIDS |
| *Cymbopogon citratus* (DC.) Stapf | Poaceae | Leaves | Cameroon: Fi [6] | 6.6 | HIV/AIDS |
| *Cynanchum viminale* subsp. *orangeanum* (Liede & Meve) Liede & Meve; Synonym: *Sarcostemma viminale* subsp.  *orangeanum* | Apocynaceae | Twigs | South Africa: Ci [12] | 3 | HIV/AIDS |

| **Botanical names** | **Families** | **Plant part used** | **Country of study: selection criteria (SC) and ethnobotanical studies (references)** | **SC index** | **Venereal diseases treated** |
| --- | --- | --- | --- | --- | --- |
| *Datura stramonium* L. | Solanaceae | Seed; fruit | Ethiopia: Fc [7]; Cameroon: Fi [6] | Fc: 1; Fi:  3.3 | Gonorrhoea |
| *Daucus carota* L. | Apiaceae | Leaves | Cameroon: Fi [6] | 2 | HIV/AIDS |
| *Dichrocephala chrysanthemifolia* (Blume) DC. | Asteraceae | Root | Ethiopia: Fc [7] | 1 | Gonorrhoea |
| *Dichrostachys cinerea* (L.) Wight & Arn. | Fabaceae | Leaves; root and stem bark | Zambia: Fi_1_ [2]; Fi_2_ [3]; RFC [4] | Fi_1_: 55; Fi_2_:  66.7; RFC:  0.2 | Gonorrhoea (Fi_2_); Syphilis (Fi_1_, Fi_2_, RFC) |
| *Dicoma anomala* Sond. | Asteraceae | Whole plant; root | South Africa: UV [18] | 0.17 | Gonorrhoea; HIV/AIDS |
| *Dioscorea alata* L. | Dioscoreaceae | Stem | Ethiopia: Fc [7] | 1 | Gonorrhoea |
| *Dioscorea cochleari-apiculata*  De Wild. | Dioscoreaceae | Root | Zambia: Fi [3] | 13.3 | Syphilis |
| *Dioscorea hirtiflora* Benth. | Dioscoreaceae | Leaves | Zambia: Fi [2] | 15 | Syphilis |
| *Dioscorea sylvatica* Eckl.; Synonyms: *Dioscorea sylvatica* var. sylvatica; *Dioscorea sylvatica* var. brevipes | Dioscoreaceae | Bulb | South Africa: Fi [14]; Ci [12] | Fi: 3.3; Ci:  3 | Gonorrhoea |
| *Diospyros lycioides* Desf. | Ebenaceae | Leaves | Zambia: Fi [2] | 45 | Gonorrhoea; Syphilis |
| *Diospyros mespiliformis*  Hochst. ex A.DC. | Ebenaceae | Stem bark | Nigeria: Fi [20] | N/A | HIV/AIDS |
| *Diospyros quiloensis* (Hiern) F.White | Ebenaceae | Stem bark | Zambia: Fi [3] | 50 | Gonorrhoea; Syphilis |
| *Diplorhynchus condylocarpon*  (Müll.Arg.) Pichon | Apocynaceae | Root | Tanzania: Ci [1] | 13 | Gonorrhoea |

| **Botanical names** | **Families** | **Plant part used** | **Country of study: selection criteria (SC) and ethnobotanical studies (references)** | **SC index** | **Venereal diseases treated** |
| --- | --- | --- | --- | --- | --- |
| *Discopodium penninervium*  Hochst. | Solanaceae | Leaves | Ethiopia: Fc [7] | 1 | Gonorrhoea |
| *Dodonaea viscosa* subsp. *angustifolia* (L.f.) J.G.West; Synonym: *Dodonaea viscosa* var. *angustifolia* | Sapindaceae | Root | South Africa: Ci [12] | 3 | HIV/AIDS; Gonorrhoea |
| *Dorstenia foetida* Schweinf. | Moraceae | Stem | Ethiopia: Fc [7] | 1 | Gonorrhoea |
| *Dracaena arborea* (Willd.) Link | Asparagaceae | Stem bark | Nigeria: Fc [11] | 63 | Gonorrhoea |
| *Dracaena fragrans* (L.) Ker Gawl.; Synonym: *Dracaena deisteliana* Engl. | Asparagaceae | Leaves | Cameroon: Fi [6] | 2 | HIV/AIDS |
| *Dracaena hyacinthoides* (L.) Mabb.; Synonym: *Sansevieria hyacinthoides* (L.) Druce | Asparagaceae | Root | South Africa: Ci [12] | 3 | HIV/AIDS |
| *Dracaena steudneri* Engl. | Asparagaceae | Stem bark | Uganda: Fm [5] | 2 | HIV/AIDS |
| *Drimia elata* Jacq. ex Willd. | Asparagaceae | Bulb | South Africa: Ci [12]; Fi: [14] | Ci: 6; Fi:  3.3 | Gonorrhoea (Fi); HIV/AIDS (Ci) |
| *Echinops amplexicaulis* Oliv. | Asteraceae | Whole root | Uganda: Fm [5] | 1 | HIV/AIDS |
| *Echinops kebericho* Mesfin | Asteraceae | Root | Ethiopia: [7] | 2 | Gonorrhoea |

| **Botanical names** | **Families** | **Plant part used** | **Country of study: selection criteria (SC) and ethnobotanical studies (references)** | **SC index** | **Venereal diseases treated** |
| --- | --- | --- | --- | --- | --- |
| *Elaeis guineensis* Jacq. | Arecaceae | Root | Nigeria: Fc [11] | 41 | Syphilis |
| *Elaeodendron transvaalense*  (Burtt Davy) R.H.Archer | Celastraceae | Root | South Africa: Ci [12] | 3 | HIV/AIDS |
| *Elephantorrhiza elephantina*  (Burch.) Skeels | Fabaceae | Root | South Africa: Ci [12] | 9 | HIV/AIDS |
| *Entada abyssinica* Steud. ex A.Rich. | Fabaceae | Leaves; stem bark; root | Uganda: Fm [5]; Ethiopia: Fc [7]; Tanzania: Ci [1] | Fm, Fc: 1;  Ci: 26.1 | HIV/AIDS |
| *Entandrophragma caudatum*  Sprague | Meliaceae | Root; fruit | Zambia: Fi [2]; RFC [4] | Fi: 25;  RFC: 0.2 | Gonorrhoea (Fi); Genital warts (Fi, RFC) |
| *Eremomastax speciosa*  (Hochst.) Cufod. | Acanthaceae | Leaves | Cameroon: Fi [6] | 2 | HIV/AIDS |
| *Erigeron bonariensis* L.; Synonym: *Conyza bonariensis* (L.) Cronquist | Asteraceae | Leaves | Uganda: Fm [5] | 1 | HIV/AIDS |
| *Erythrina abyssinica* DC. | Fabaceae | Stem bark | Uganda: Fm [5] | 5 | HIV/AIDS |
| *Erythrina brucei* Schweinf. | Fabaceae | Stem bark | Ethiopia: Fc [7] | 2 | Gonorrhoea |
| *Erythrina senegalensis* DC. | Fabaceae | Stem bark | Nigeria: Fi [20] | N/A | HIV/AIDS |
| *Erythrococca bongensis* Pax | Euphorbiaceae | Leaves | Uganda: Fm [5] | 1 | HIV/AIDS |
| *Erythrophleum africanum*  (Benth.) Harms | Fabaceae | Leaves | Namibia: Fi [19] | 18 | Gonorrhoea |
| *Erythrophleum suaveolens*  (Guill. & Perr.) Brenan | Fabaceae | Stem bark | Nigeria: UMi [16] | 18.8 | Gonorrhoea; Syphilis |

| **Botanical names** | **Families** | **Plant part used** | **Country of study: selection criteria (SC) and ethnobotanical studies (references)** | **SC index** | **Venereal diseases treated** |
| --- | --- | --- | --- | --- | --- |
| *Eucalyptus saligna* Sm. | Myrtaceae | Leaves | Cameroon: Fi [6] | 6.6 | HIV/AIDS |
| *Eucalyptus* sp. | Myrtaceae | Leaves; stem bark | Uganda: Fm [5] | 3 | HIV/AIDS |
| *Eucalyptus* spp. | Myrtaceae | Bark | South Africa: UV [18] | 0.04 | Gonorrhoea |
| *Euclea coriacea* A*.*DC*.* | Ebenaceae | Root | South Africa: UV [18] | 0.04 | Gonorrhoea |
| *Euclea crispa* subsp. *crispa* | Ebenaceae | Root | South Africa: Ci [12] | 6 | HIV/AIDS |
| *Euclea divinorum* Hiern | Ebenaceae | Leaves and stem; root | Zambia: Fi_1_ [2]; Fi_2_ [3]; Ethiopia: Fc [7] | Fi_1_: 35; Fi_2_:  20; Fc: 1 | Gonorrhoea (Fc, Fi_2_); Genital herpes (Fi_1_, Fi_2_); Syphilis (Fi_1_, Fi_2_) |
| *Euclea racemosa* L. | Ebenaceae | Root | Ethiopia: Fc [7] | 2 | Gonorrhoea |
| *Eucomis pallidiflora* subsp. *pole-evansii* (N.E.Br.) Reyneke ex J.C.Manning | Asparagaceae | Whole plant | South Africa: Ci [12] | 3 | Chlamydia |
| *Euphorbia abyssinica*  J.F.Gmel. | Euphorbiaceae | Stem bark | Ethiopia: Fc [7] | 1 | Gonorrhoea |
| *Euphorbia ampliphylla* Pax | Euphorbiaceae | Latex | Ethiopia: Fc [7] | 1 | Gonorrhoea |
| *Euphorbia benthamii* Hiern | Euphorbiaceae | Leaves | Zambia: Fi [2]; RFC [4] | Fi: 20;  RFC: 0.2 | Gonorrhea (Fi, RFC); Chlamydia (Fi) |
| *Euphorbia cactus* Ehrenb. ex Boiss. | Euphorbiaceae | Latex | Ethiopia: Fc [7] | 1 | Gonorrhoea |

| **Botanical names** | **Families** | **Plant part used** | **Country of study: selection criteria (SC) and ethnobotanical studies (references)** | **SC index** | **Venereal diseases treated** |
| --- | --- | --- | --- | --- | --- |
| *Euphorbia candelabrum*  Trémaux ex Kotschy | Euphorbiaceae | Latex | Ethiopia: Fc [7] | 2 | Gonorrhoea |
| *Euphorbia depauperata*  Hochst. ex A.Rich. | Euphorbiaceae | Root | Ethiopia: Fc [7] | 1 | Gonorrhoea |
| *Euphorbia dumalis* S.Carter | Euphorbiaceae | Root and stem bark | Ethiopia: Fc [7] | 1 | Gonorrhoea |
| *Euphorbia grantii* Oliv. | Euphorbiaceae | Root | Tanzania: Ci [1] | 13 | Gonorrhoea |
| *Euphorbia lateriflora*  Schumach. & Thonn. | Euphorbiaceae | Stem | Nigeria: Fu [10]; UMi [16] | Fu: 2;  UMi: 25 | Gonorrhoea (Fu); Syphilis (Fu, UMi) |
| *Euphorbia lathyris* L. | Euphorbiaceae | Seed | Ethiopia: Fc [7] | 1 | Gonorrhoea |
| *Euphorbia longituberculosa*  Hochst. ex Boiss. | Euphorbiaceae | Stem | Ethiopia: Fc [7] | 1 | Gonorrhoea |
| *Euphorbia maleolens* E.Phillips | Euphorbiaceae | Whole plant | South Africa: Ci [12] | 12 | HIV/AIDS |
| *Euphorbia piscidermis*  M.G.Gilbert | Euphorbiaceae | Root and seed | Ethiopia: Fc [7] | 1 | Gonorrhoea |
| *Euphorbia schimperiana*  Scheele | Euphorbiaceae | Root | Ethiopia: Fc [7] | 1 | Gonorrhoea |
| *Euphorbia stapfii* A.Berger | Euphorbiaceae | Sap | Uganda: Fm [5] | 1 | HIV/AIDS |
| *Euploca ovalifolia* (Forssk.) Diane & Hilger; Synonym: *Heliotropium ovalifolium* Forssk. | Boraginaceae | Root | Nigeria: Fi [20] | N/A | HIV/AIDS |

| **Botanical names** | **Families** | **Plant part used** | **Country of study: selection criteria (SC) and ethnobotanical studies (references)** | **SC index** | **Venereal diseases treated** |
| --- | --- | --- | --- | --- | --- |
| *Fagaropsis angolensis* (Engl.) H.M.Gardner | Rutaceae | Leaves | Ethiopia: Fc [7] | 1 | Gonorrhoea |
| *Faidherbia albida* (Delile) A.Chev.; Synonym: *Acacia albida* Delile | Fabaceae | Leaves and stem bark | Zambia: Fi [3] | 50 | Syphilis |
| *Ficus exasperata* Vahl | Moraceae | Leaves and root | Nigeria: Fc [11] | 12 | Gonorrhoea |
| *Ficus mucuso* Welw. ex Ficalho | Moraceae | Leaves | Uganda: Fm [5] | 1 | HIV/AIDS |
| *Ficus natalensis* Hochst. | Moraceae | Leaves | Uganda: Fm [5]; Zambia: Fi [3] | Fm: 15; Fi:  1 | Genital warts (Fm); HIV/AIDS (Fi) |
| *Ficus polita* Vahl | Moraceae | Bark | Nigeria: Fi [17] | 2 | Gonorrhoea |
| *Ficus sur* Forssk.; Synonym:  *Ficus capensis* Thunb. | Moraceae | Leaves | Zambia: Fi [3] | 60 | Genital warts |
| *Ficus thonningii* Blume | Moraceae | Stem bark | Nigeria: Fi [20] | N/A | HIV/AIDS |
| *Ficus vallis-choudae* Delile | Moraceae | Stem bark | Nigeria: Fi [20] | N/A | HIV/AIDS |
| *Flacourtia indica* (Burm.f.) Merr. | Salicaceae | Root bark | Ethiopia: Fc [7] | 1 | Gonorrhoea |
| *Flueggea virosa* (Roxb. ex Willd.) Royle | Phyllanthaceae | Stem bark; whole root | Uganda: Fm [5]; Botswana: Fi [13] | Fm: 2; Fi:  N/A | HIV/AIDS (Fm); Genital herpes (Fi) |
| *Foeniculum vulgare* Mill. | Apiaceae | Leaves; root and stem | Cameroon: Fi [6]; Ethiopia: Fc [7] | Fi: 2; Fc: 5 | Gonorrhoea (Fc); HIV/AIDS (Fi) |
| *Garcinia livingstonei*  T.Anderson | Clusiaceae | Fruit | Zambia: Fi [3] | 83.3 | Genital herpes |
| *Geigeria aspera* Harv.;  Synonym: *Geigeria aspera* var. aspera | Asteraceae | Whole plant | South Africa: Ci [12] | 3 | HIV/AIDS |

| **Botanical names** | **Families** | **Plant part used** | **Country of study: selection criteria (SC) and ethnobotanical studies (references)** | **SC index** | **Venereal diseases treated** |
| --- | --- | --- | --- | --- | --- |
| *Gethyllis namaquensis*  (Schönland) Oberm. | Amaryllidaceae | Bulb | South Africa: Ci [12] | 3 | Chlamydia |
| *Gladiolus dalenii* Van Geel | Iridaceae | Root | Ethiopia: Fc [7]; Nigeria: Fu [10]; UMi [16] | Fc: 1; Fu:  3; UMi:  62.5 | Gonorrhoea |
| *Glyphaea brevis* (Spreng.) Monach | Malvaceae | Leaves; root | Nigeria: Fu [10]; UMi [16] | Fu: 2;  UMi: 25 | Gonorrhoea |
| *Gnidia stenophylla* Gilg | Thymelaeaceae | Root | Ethiopia: Fc [7] | 1 | Gonorrhoea |
| *Gomphocarpus integer*  (N.E.Br.) Bullock | Apocynaceae | Root | Ethiopia: [7] | 2 | Gonorrhoea |
| *Gomphocarpus tomentosus*  Burch | Apocynaceae | Leaves and stem | Namibia: Fi_1_, Fi_2_ [19] | Fi_1_: 18; Fi_2_:  14 | Gonorrhoea (Fi_1_); Syphilis (Fi_2_) |
| *Gossypium hirsutum* L. | Malvaceae | Leaves | Nigeria: Fc [11] | 44 | Gonorrhoea |
| *Grewia pubescens* P.Beauv. | Malvaceae | Root bark | Uganda: Fm [5] | 1 | HIV/AIDS |
| *Grewia villosa* Willd. | Malvaceae | Leaves | Ethiopia: Fc [7] | 1 | Gonorrhoea |
| *Guibourtia tessmannii* (Harms)  J. Léonard | Fabaceae | Stem bark | Cameroon: Fi [6] | 4.6 | HIV/AIDS |
| *Guizotia scabra* Chiov. | Asteraceae | Leaves; stem bark; flower; fruit | Uganda: Fm [5] | 1 | HIV/AIDS |
| *Gymnanthemum amygdalinum* (Delile) Sch.Bip.; Synonym: *Vernonia amygdalina* Delile | Asteraceae | Leaves; root wood | Uganda: Fm [5] | 2 | HIV/AIDS |
| *Gymnosporia senegalensis* Loes.; Synonym: *Maytenus senegalensis* (Lam.) Exell | Celastraceae | Root bark; root wood; stem bark; leaves | Uganda: Fm [5] | 3 | HIV/AIDS |

| **Botanical names** | **Families** | **Plant part used** | **Country of study: selection criteria (SC) and ethnobotanical studies (references)** | **SC index** | **Venereal diseases treated** |
| --- | --- | --- | --- | --- | --- |
| *Harpagophytum procumbens*  (Burch.) DC. ex Meisn. | Pedaliaceae | Fruit | Botswana: Fi [13] | N/A | HIV/AIDS |
| *Harrisonia abyssinica* Oliv. | Rutaceae | Root | Tanzania: Ci [1] | 17.4 | HIV/AIDS |
| *Helianthus annuus* L. | Asteraceae | Leaves; seeds | Cameroon: Fi [6] | 6.6 | HIV/AIDS |
| *Helichrysum caespititium* (DC.) Sond.  . | Asteraceae | Whole plant | South Africa: Ci [12]; Fi [14] | Fi: 3.3; Ci:  3 | Gonorrhoea |
| *Helichrysum nudifolium* var.  *oxyphyllum* (DC.) Beentje | Asteraceae | Whole root | Uganda: Fm [5] | 1 | HIV/AIDS |
| *Helinus integrifolius* Kuntze | Rhamnaceae | Root | Zambia: Fi [2] | 15 | Gonorrhoea; Syphilis |
| *Hermannia depressa* N*.*E*.*Br*.* | Malvaceae | Root | South Africa: UV [18] | 0.08 | Gonorrhoea |
| *Heteromorpha arborescens* var. *abyssinica* (Hochst. ex A.Rich.) H.Wolff; Synonym: *Heteromorpha trifoliata* Eckl. &amp; Zeyh*.* | Apiaceae | Root | South Africa: UV [18] | 0.04 | Gonorrhoea |
| *Hibiscus rosa-sinensis* L. | Malvaceae | Leaves | Cameroon: Fi [6] | 2.6 | HIV/AIDS |
| *Hibiscus rostellatus* Guill. & Perr. | Malvaceae | Whole plant | Nigeria: Fi [20] | N/A | HIV/AIDS |
| *Hibiscus surattensis* L. | Malvaceae | Leaves | Nigeria: Fc [11] | 70 | Gonorrhoea |
| *Holarrhena pubescens* Wall. & G.Don | Apocynaceae | Root | Tanzania: Ci [1] | 13 | Syphilis |

| **Botanical names** | **Families** | **Plant part used** | **Country of study: selection criteria (SC) and ethnobotanical studies (references)** | **SC index** | **Venereal diseases treated** |
| --- | --- | --- | --- | --- | --- |
| *Hydrocotyle mannii* Hook.f. | Araliaceae | Whole plant | Uganda: Fm [5] | 1 | HIV/AIDS |
| *Hygrophila auriculata*  (Schumach.) Heine | Acanthaceae | Leaves | Uganda: Fm [5] | 1 | HIV/AIDS |
| *Hymenocardia acida* Tul. | Phyllanthaceae | Stem bark | Uganda: Fm [5] | 1 | HIV/AIDS |
| *Hypoxis hemerocallidea* Fisch., C.A.Mey. & Avé-Lall. | Hypoxidaceae | Tuber | South Africa: Ci [12]; Fi [14] | Ci: 12; Fi:  6.7 | Gonorrhoea (Fi); HIV/AIDS (Ci) |
| *Hypoxis obtusa* Burch. | Hypoxidaceae | Tuber | South Africa: Ci [12] | 3 | Chlamydia |
| *Impatiens ethiopica* Grey- Wilson | Balsaminaceae | Root | Ethiopia: Fc [7] | 1 | Gonorrhoea |
| *Indigofera ormocarpoides*  Baker | Fabaceae | Leaves | Zambia: Fi [2] | 25 | Syphilis |
| *Ipomoea cicatricosa* Baker | Convolvulaceae | Root | Ethiopia: Fc [7] | 1 | Gonorrhoea |
| **Ipomoea hildebrandtii* Vatke | Convolvulaceae | Leaves | Uganda: Fm [5] | 1 | HIV/AIDS |
| *Ipomoea obscura* var. *obscura* | Convolvulaceae | Root | South Africa: Ci [12]; Fi [14] | Ci: 1; Fi:  3.3 | Gonorrhoea |
| *Ipomoea verbascoidea* Choisy | Convolvulaceae | Root | Zambia: Fi [2] | 25 | Gonorrhoea |
| *Ipomoea wightii* Choisy | Convolvulaceae | Sap | Uganda: Fm [5] | 1 | HIV/AIDS |
| *Ixora coccinea* L. | Rubiaceae | Whole plant | Nigeria: Fc [11] | 23 | Gonorrhoea |
| *Jacaranda mimosifolia* D.Don | Bignoniaceae | Leaves; stem bark | South Africa: Fu [9] | 8 | Gonorrhoea |
| *Jatropha curcas* L. | Euphorbiaceae | Leaves; root | Nigeria: Fc [11]; Fi_1_ [17]; Fi_2_ [20] | Fc_1_: 30;  Fc_2_: 12;  Fi_1_: 8; Fi_2_:  13 | Gonorrhoea (Fc_1_, Fi_1_); Syphilis (Fc_2_); HIV/AIDS (Fi_2_) |

| **Botanical names** | **Families** | **Plant part used** | **Country of study: selection criteria (SC) and ethnobotanical studies (references)** | **SC index** | **Venereal diseases treated** |
| --- | --- | --- | --- | --- | --- |
| *Jatropha zeyheri* Sond. | Euphorbiaceae | Root | South Africa: Ci [12]; Fi [14] | Ci: 3; Fi:  3.3 | Gonorrhoea |
| *Justicia flava* (Forssk.) Vahl | Acanthaceae | Leaves | Uganda: Fm [5] | 1 | HIV/AIDS |
| *Justicia schimperiana* subsp. *schimperiana*; Synonym: *Adhatoda schimperiana* Hochst. ex Nees | Acanthaceae | Leaves | Ethiopia: Fc [7] | 1 | Gonorrhoea |
| *Justicia schimperiana*  T.Anderson | Acanthaceae | Leaves and root | Ethiopia: Fc [7] | 2 | Gonorrhoea |
| *Kalaharia uncinata* (Schinz) Moldenke; Synonym: *Clerodendrum uncinatum* Schinz | Lamiaceae | Root | Zambia: Fi [2] | 30 | Gonorrhoea |
| *Kalanchoe delagoensis* Eckl. & Zeyh. Synonym: *Bryophyllum delagoense* (Eckl. & Zeyh.) Shinz | Crassulaceae | Leaves | South Africa: Fu [9] | 5 | Gonorrhoea |
| *Kalanchoe densiflora* Rolfe | Crassulaceae | Leaves | Ethiopia: Fc [7] | 4 | Gonorrhoea |
| *Kalanchoe pinnata* (Lam.) Pers.; Synonym: *Bryophyllum pinnatum* (Lam.) Oken | Crassulaceae | Leaves | Uganda: Fm [5] | 1 | HIV/AIDS |
| *Kigelia africana* (Lam.) Benth. | Bignoniaceae | Fruit; stem bark and leaves; root bark | Zambia: Fi1 [2]; Fi2 [3]; RFC [4]; Uganda: Fm [5] | Fi1: 75; Fi2:  83.3; Fm:  1; RFC: 0.8 | Syphilis (Fi1, Fi2, Fm, RFC); HIV/AIDS, Genital herpes (RFC) |
| *Kleinia longiflora* DC. | Asteraceae | Root | South Africa: Ci [12] | 3 | Chlamydia |
| *Kniphofia isoetifolia* A.Rich. | Asphodelaceae | Root | Ethiopia: Fc [7] | 1 | Gonorrhoea |

| **Botanical names** | **Families** | **Plant part used** | **Country of study: selection criteria (SC) and ethnobotanical studies (references)** | **SC index** | **Venereal diseases treated** |
| --- | --- | --- | --- | --- | --- |
| *Lagenaria breviflora* (Benth.) Roberty | Cucurbitaceae | Fruit | Nigeria: Fu [10] | 1 | Gonorrhoea; Syphilis |
| *Lagenaria siceraria* (Molina) Standl. | Cucurbitaceae | Fruit and seed | Ethiopia: Fc [7] | 2 | Gonorrhoea |
| *Lannea discolor* Engl. | Anacardiaceae | Fruit pulp | Zambia: Fi [3] | 50 | Gonorrhoea |
| *Lannea edulis* Engl. | Anacardiaceae | Root | Botswana: Fi [13] | N/A | HIV/AIDS |
| *Lannea schweinfurthii* var. *stuhlmannii* (Engl.) Kokwaro; Synonym: *Lannea stuhlmannii* (Engl.) Eyles | Anacardiaceae | Leaves; root | Zambia: Fi_1_ [2]; Fi_2_ [3] | Fi_1_: 83.3;  Fi_2_: 25 | Gonorrhoea (Fi_1_, Fi_2_); Syphilis (Fi_1_, Fi_2_); Genital herpes (Fi_1_); HIV/AIDS (Fi_1_) |
| *Lantana camara* L. | Verbenaceae | Leaves; twigs; root | South Africa: FL_1_, FL_2_, FL_3_, FL_4_ [8]; Ethiopia: Fc [7] | Fc: 1; FL_1_: 50; FL_2_, FL_3_, FL_4_: 16.7 | Gonorrhoea (Fc, FL_1_); Chlamydia (FL_2_); Syphilis (FL_3_); HIV/AIDS (FL_4_) |
| *Lantana trifolia* L. | Verbenaceae | Root | Ethiopia: Fc [7] | 1 | Gonorrhoea |
| *Lasianthera africana* P.Beauv. | Stemonuraceae | Leaves | Nigeria: Fc [11] | 26 | Gonorrhoea |
| *Ledebouria cooperi* (Hook.f.) Jessop | Hyacinthaceae | Root | Botswana: Fi [13] | N/A | Genital herpes |
| *Leea guineensis* G.Don | Leeaceae | Leaves | Cameroon: Fi [6] | 3.3 | HIV/AIDS |
| *Leonotis nepetifolia* (L.) R.Br. | Lamiaceae | Leaves | Uganda: Fm [5] | 2 | HIV/AIDS |
| *Lepidium sativum* L. | Brassicaceae | Leaves | Ethiopia: Fc [7] | 1 | Gonorrhoea |

| **Botanical names** | **Families** | **Plant part used** | **Country of study: selection criteria (SC) and ethnobotanical studies (references)** | **SC index** | **Venereal diseases treated** |
| --- | --- | --- | --- | --- | --- |
| *Leplaea thompsonii* (Sprague & Hutch.) E.J.M.Koenen & J.J.de Wilde; Synonym: *Guarea thompsonii* Sprague & Hutch. | Meliaceae | Stem | Nigeria: Fc [11] | 26 | Gonorrhoea |
| *Leptadenia* sp. | Apocynaceae | Root | Ethiopia: Fc [7] | 1 | Gonorrhoea |
| *Litogyne gariepina* (DC.) Anderb.; Synonym: *Epaltes elata* Steetz | Asteraceae | Leaves | Namibia: Fi [19] | 93 | Syphilis |
| *Lonchocarpus capassa* Rolfe | Fabaceae | Stem bark and leaves | Zambia: Fi [3] | 13.3 | Gonorrhoea |
| *Maesa lanceolata* Forssk. | Primulaceae | Whole root; leaves; root wood | Uganda: Fm [5] | 3 | HIV/AIDS |
| *Mangifera indica* L. | Anacardiaceae | Leaves; stem bark | Nigeria: Fc_1_, Fc_2_ [11]; UMi [16]; Uganda: Fm [5] | Fc_1_: 34;  Fc_2_: 18;  Fm: 4;  UMi: 26.3 | Gonorrhoea (Fc_1_); Syphilis (Fc_2_, UMi); HIV/AIDS (Fm) |
| *Manihot esculenta* Crantz | Euphorbiaceae | Leaves | Cameroon: Fi [6] | 2 | HIV/AIDS |
| *Mansonia altissima* (A.Chev.) A.Chev. | Sterculiaceae | Stem bark | Cameroon: Fi [6] | 2 | HIV/AIDS |
| *Marantochloa cuspidata*  (Roscoe) Milne-Redh. | Marantaceae | Leaves | Nigeria: Fc [11] | Fc1: 16;  Fc_2_: 10 | Gonorrhoea (Fc1); Syphilis (Fc_2_) |
| *Marantochloa leucantha*  (K.Schum.) Milne-Redh. | Marantaceae | Leaves and root | Ethiopia: Fc [7] | 1 | Gonorrhoea |
| *Markhamia lutea* (Benth.) K.Schum. | Bignoniaceae | Root bark | Uganda: Fm [5] | 1 | HIV/AIDS |

| **Botanical names** | **Families** | **Plant part used** | **Country of study: selection criteria (SC) and ethnobotanical studies (references)** | **SC index** | **Venereal diseases treated** |
| --- | --- | --- | --- | --- | --- |
| *Melia azedarach* L. | Meliaceae | Stem bark; whole plant | South Africa: Fu [9] | 12 | Gonorrhoea |
| *Mentha longifolia* (L.) Huds. | Lamiaceae | Root | South Africa: Fu [9] | 3 | Gonorrhoea |
| *Mentha* sp.* | Lamiaceae | Leaves | Uganda: Fm [5] | 1 | HIV/AIDS |
| *Microdesmis puberula* Hook.f. | Pandaceae | Leaves | Nigeria: Fc [11] | 37 | Gonorrhoea |
| *Microglossa pyrifolia* (Lam.) Kuntze | Asteraceae | Whole root | Uganda: Fm [5] | 1 | HIV/AIDS |
| *Mimosa pigra* L. | Fabaceae | Whole plant | Zambia: Fi [3] | 83.3 | Gonorrhoea |
| *Mitragyna inermis* (Willd.) Kuntze | Rubiaceae | Root | Nigeria: Fi [17] | 2 | Chlamydia |
| *Momordica balsamina* L. | Cucurbitaceae | Whole plant | Zambia: Fi [2]; RFC [4] | Fi: 50;  RFC: 0.4 | Gonorrhoea (Fi); Syphilis (Fi, RFC); HIV/AIDS (Fi) |
| *Momordica boivinii* Baill. | Cucurbitaceae | Root | Ethiopia: Fc [7] | 1 | Gonorrhoea |
| *Momordica charantia* L. | Cucurbitaceae | Leaves | Nigeria: UMi [16] | 50 | Gonorrhoea; Syphilis; Trichomoniasis |
| *Momordica foetida* Schumach. | Cucurbitaceae | Leaves and root | Ethiopia: Fc [7] | 2 | Gonorrhoea |
| *Monanthotaxis obovata* (Benth.) P.H.Hoekstra; Synonym: *Friesodielsia obovata* (Benth.) Verdc. | Annonaceae | Root | Tanzania: Ci [1] | 21.7 | Gonorrhoea; Syphilis |
| *Morinda lucida* Benth | Rubiaceae | Leaves | Nigeria: UMi [16] | 31.3 | Trichomoniasis |

| **Botanical names** | **Families** | **Plant part used** | **Country of study: selection criteria (SC) and ethnobotanical studies (references)** | **SC index** | **Venereal diseases treated** |
| --- | --- | --- | --- | --- | --- |
| *Moringa oleifera* Lam. | Moringaceae | Leaves; fruit; seeds; root | Cameroon: Fi_1_ [6]; Uganda: Fm [5]; Nigeria: Fi_2_ [17] | Fi_1_: 10.5;  Fi_2_: 5; Fm:  1 | HIV/AIDS (Fi_1_, Fm);  Gonorrhoea (Fi_2_) |
| *Musa paradisiaca* L.; Synonym: *Musa sapientum* L. | Musaceae | Leaves; corm | Uganda: Fm [5]; Kenya: ICf [15] | Fm: 1; ICf:  0.4 | Gonorrhoea (ICf); HIV/AIDS (Fm) |
| *Myrica kandtiana* Engl.; Synonym: *Morella kandtiana* (Engl.) Verdc. & Polhill | Myricaceae | Whole root; stem bark; stem wood | Uganda: Fm [5] | 3 | HIV/AIDS |
| *Myrothamnus flabellifolia*  Welw. | Myrothamnaceae | Whole plant | South Africa: Ci [12] | 3 | HIV/AIDS |
| *Nauclea latifolia* Sm.; Synonym: *Sarcocephalus latifolius* (Sm) E.A.Bruce | Rubiaceae | Root; root bark; whole root; tuber | Nigeria: Fc [11]; Uganda: Fm [5] | Fc: 29; Fm:  5 | Gonorrhoea (Fc); HIV/AIDS (Fm) |
| *Nicoteba betonica* (L.) Lindau; Synonym: *Justicia betonica* L. | Acanthaceae | Leaves | Uganda: Fm [5] | 1 | HIV/AIDS |
| *Nicotiana glauca* Graham | Solanaceae | Root | South Africa: FL [8] | 50 | Gonorrhoea; Syphilis |
| *Nicotiana tabacum* L. | Solanaceae | Leaves; root | Ethiopia: Fc [7]; Nigeria: Fu [10] | Fc: 1; Fu: 2 | Gonorrhoea (Fc, Fu); HIV/AIDS (Fu) |
| *Ocimum basilicum* L. | Lamiaceae | Leaves | Cameroon: Fi [6] | 2 | HIV/AIDS |
| *Ocimum gratissimum* L. | Lamiaceae | Leaves | Nigeria: Fu [10] | 3 | Gonorrhoea |
| *Ocimum gratissimum* subsp. *gratissimum* Synonym: *Ocimum suave* Willd. | Lamiaceae | Leaves | Uganda: Fm [5] | 1 | HIV/AIDS |
| *Olax subscorpioidea* Oliv. | Olacaceae | Root | Nigeria: UMi [16] | 37.5 | Syphilis; Trichomoniasis |

| **Botanical names** | **Families** | **Plant part used** | **Country of study: selection criteria (SC) and ethnobotanical studies (references)** | **SC index** | **Venereal diseases treated** |
| --- | --- | --- | --- | --- | --- |
| *Oldfieldia dactylophylla*  (Welw. ex Oliv.) J.Léonard | Picrodendraceae | Root | Tanzania: Ci [1] | 13 | Gonorrhoea |
| *Olea europaea* L. | Oleaceae | Seed | Uganda: Fm [5] | 1 | HIV/AIDS |
| *Olea europaea* subsp.  *cuspidata* (Wall. & G.Don) Cif. | Oleaceae | Stem bark | Ethiopia: Fc [7] | 1 | Gonorrhoea |
| *Opuntia ficus-indica* (L.) Mill. | Cactaceae | Root | South Africa: Ci [12]; Fi [14]; FL_1_, FL_2_, FL_3_, FL_4_  [8] | Ci: 6; Fi:  6.7; FL_1_:  63.6; FL_2_, FL_3_, FL_4_: 9.1 | Gonorrhoea (Ci, Fi, FL_1_); Chlamydia (FL_2_); Syphilis (FL_3_); Genital warts (FL_4_) |
| *Opuntia stricta* (Haw.) Haw. | Cactaceae | Root | South Africa: FL [8] | 50 | Gonorrhoea; Genital warts |
| *Oreosyce africana* Hook.f. | Cucurbitaceae | Root | Ethiopia: Fc [7] | 1 | Gonorrhoea |
| *Ozoroa insignis* Delile | Anacardiaceae | Leaves | Ethiopia: Fc [7] | 1 | Gonorrhoea |
| *Palisota hirsuta* K.Schum. | Commelinaceae | Leaves | Nigeria: Fc [11] | 61 | Gonorrhoea |
| *Pelargonium* spp. | Geraniaceae | Root | South Africa: Ci [12] | 3 | HIV/AIDS |
| *Peltophorum africanum* Sond. | Caesalpiniaceae | Root; stem bark, leaves | South Africa: Ci [12]; Zambia: RFC [4] | Ci: 6; RFC:  0.4 | HIV/AIDS (Ci);  Gonorrhoea, Syphilis (RFC) |
| *Pentanisia prunelloides*  (Klotzsch) Walp. | Rubiaceae | Whole plant | South Africa: UV [18] | 0.33 | Gonorrhoea |
| *Periploca linearifolia* Quart.- Dill. & A.Rich. | Apocynaceae | Latex | Ethiopia: Fc [7] | 1 | Gonorrhoea |
| *Persea americana* Mill. | Lauraceae | Seed | Uganda: Fm [5] | 1 | HIV/AIDS |

| **Botanical names** | **Families** | **Plant part used** | **Country of study: selection criteria (SC) and ethnobotanical studies (references)** | **SC index** | **Venereal diseases treated** |
| --- | --- | --- | --- | --- | --- |
| *Petroselinum crispum* (Mill.) Fuss | Apiaceae | Leaves | Cameroon: Fi [6] | 2 | HIV/AIDS |
| *Phaseolus vulgaris* L. | Fabaceae | Seed | Botswana: Fi [13] | N/A | Genital herpes |
| *Philenoptera cyanescens* (Schumach. & Thonn.) Roberty; Synonym: *Lonchocarpus cyanescens* (Schumach. & Thonn.) Benth. | Fabaceae | Leaves and bark | Nigeria: Fc [11]; Fi [20] | 41 | Syphilis |
| *Philenoptera laxiflora* (Guill. & Perr.) Roberty; Synonym: *Lonchocarpus laxiflorus* Guill. & Perr. | Papilionaceae | Root bark | Uganda: Fm [5] | 1 | HIV/AIDS |
| *Phyllanthus engleri* Pax | Phyllanthaceae | Root | Tanzania: Ci [1] | 17.4 | Syphilis |
| *Phyllanthus reticulatus* Poir. | Phyllanthaceae | Leaves | Zambia: Fi [3] | 26.7 | Genital herpes |
| *Phytolacca dodecandra* L'Hér. | Phytolaccaceae | Leaves and root | Ethiopia: Fc [7] | 9 | Gonorrhoea |
| *Piliostigma reticulatum* (DC.) Hochst. | Fabaceae | Bark | Nigeria: Fi [17] | 3 | Syphilis |
| *Piliostigma thonningii*  (Schumach.) Milne-Redh. | Fabaceae | Root bark; stem bark and root | Zambia: Fi [3]; Uganda: Fm [5] | Fi: 60; Fm:  3 | Syphilis (Fi); HIV/AIDS (Fm) |
| *Piper guineense* Thonn. | Piperaceae | Seeds | Nigeria: Fc [11] | Fc_1_: 32;  Fc_2_: 15 | Gonorrhoea (Fc_1_); Syphilis (Fc_2_) |
| *Piptadeniastrum africanum*  (Hook.f.) Brenan | Fabaceae | Stem bark | Uganda: Fm [5] | 1 | HIV/AIDS |
| *Plectranthus barbatus* Andrews | Lamiaceae | Leaves | Uganda: Fm [5] | 3 | HIV/AIDS |

| **Botanical names** | **Families** | **Plant part used** | **Country of study: selection criteria (SC) and ethnobotanical studies (references)** | **SC index** | **Venereal diseases treated** |
| --- | --- | --- | --- | --- | --- |
| *Plectranthus ciliatus* E.Mey. ex Benth. | Lamiaceae | Root | South Africa: Ci [12] | 3 | HIV/AIDS |
| *Plumbago zeylanica* L. | Plumbaginaceae | Leaves; root | Uganda: Fm [5]; Ethiopia: Fc [7]; Nigeria: Fu [10] | Fm: 1; Fc:  2; Fu: 4 | HIV/AIDS (Fm);  Gonorrhoea (Fc, Fu) |
| *Polyscias fulva* (Hiern) Harms | Araliaceae | Stem bark | Uganda: Fm [5] | 1 | HIV/AIDS |
| *Portulaca oleracea* L. | Portulacaceae | Whole plant | Nigeria: Fc [11] | Fc_1_: 12;  Fc_2_: 9 | Gonorrhoea (Fc_1_); Syphilis (Fc_2_) |
| *Premna senensis* Klotzsch | Lamiaceae | Leaves | Zambia: Fi [3] | 13.3 | Syphilis |
| **Protea caffra* subsp. *caffra*; Synonym: *Protea caffra* Meisn. | Proteaceae | Seed | South Africa: Ci [12] | 3 | Chlamydia |
| *Prunus africana* (Hook.f.) Kalkaman | Rosaceae | Stem bark; root; root bark; root wood | Ethiopia: Fc [7]; Uganda: Fm [5]; Kenya: ICf [15] | Fc: 3; Fm:  1; ICf: 0.6 | Gonorrhoea (Fc); HIV/AIDS (Fm, ICf) |
| *Pseudocedrela kotschyi* Harms | Meliaceae | Root bark; root wood | Uganda: Fm [5] | 1 | HIV/AIDS |
| *Psidium guajava* L. | Myrtaceae | Leaves; stem bark | South Africa: FL [8]; Uganda: Fm [5] | FL_1_: 66.7;  FL_2_: 33.3;  Fm: 3 | Gonorrhoea (FL_1_); Genital warts (FL_2_); HIV/AIDS (Fm) |
| *Psorospermum febrifugum*  Spach | Hypericaceae | Stem bark; whole root; root bark | Uganda: Fm [5] | 5 | HIV/AIDS |
| *Raphanus raphanistrum* subsp. *sativus* (L.) Dominic; Synonym: *Raphanus sativus* L. | Brassicaceae | Leaves | Cameroon: Fi [6] | 2 | HIV/AIDS |
| *Rauvolfia vomitoria* Wennberg | Apocynaceae | Leaves; whole root | Nigeria: Fc [11]; Uganda: Fm [5] | Fc: 16; Fm:  1 | Gonorrhoea (Fc); HIV/AIDS (Fm) |

| **Botanical names** | **Families** | **Plant part used** | **Country of study: selection criteria (SC) and ethnobotanical studies (references)** | **SC index** | **Venereal diseases treated** |
| --- | --- | --- | --- | --- | --- |
| *Rhigozum brevispinosum*  Kuntze | Bignoniaceae | Whole plant | Namibia: Fi [19] | 32 | Syphilis |
| *Ricinus communis* L. | Euphorbiaceae | Root; leaves; fruit; whole plant | South Africa: FL [8]; Fu [9]; Ethiopia: Fc [7] | FL_1_: 33.3;  FL_2_: 22.2;  FL_3_: 11.1;  Fu: 11; Fc:  1 | Gonorrhoea (FL_1_, Fu, Fc); Chlamydia (FL_2_); Syphilis (FL_3_) |
| *Rotheca myricoides* (Hochst.) Steane & Mabb.; Synonym: *Clerodendrum myricoides* (Hochst.) R.Br. & Vatke | Lamiaceae | Whole root; root wood | Tanzania: Ci [1]; Uganda: Fm [5] | Ci: 30.4;  Fm: 3 | Gonorrhoea (Ci); Syphilis (Ci); HIV/AIDS (Fm) |
| *Rubia cordifolia* L. | Rubiaceae | Root | Ethiopia: Fc [7] | 1 | Gonorrhoea |
| *Rubus steudneri* Schweinf. | Rosaceae | Leaves | Ethiopia: Fc [7] | 1 | Gonorrhoea |
| *Rumex abyssinicus* Jacq. | Polygonaceae | Rot | Ethiopia: Fc [7] | 1 | Gonorrhoea |
| *Rumex nepalensis* Spreng. | Polygonaceae | Root | Ethiopia: Fc [7] | 1 | Gonorrhoea |
| *Ruta chalepensis* L. | Rutaceae | Leaves | Ethiopia: Fc [7] | 1 | Gonorrhoea |
| *Sabicea calycina* Benth. | Rubiaceae | Stem bark | Nigeria: UMi [16] | 43.8 | Syphilis |
| *Sclerocarya birrea* Hochst.; Synonym: *Sclerocarya birrea* subsp. *birrea* | Anacardiaceae | Bark | South Africa: Ci [12] | 3 | HIV/AIDS |
| *Sclerocarya birrea* subsp. *caffra* (Sond.) Kokwaro; Synonym: *Sclerocarya caffra*  Sond. | Anacardiaceae | Leaves | Zambia: Fi [2] | 30 | Gonorrhoea; HIV/AIDS |

| **Botanical names** | **Families** | **Plant part used** | **Country of study: selection criteria (SC) and ethnobotanical studies (references)** | **SC index** | **Venereal diseases treated** |
| --- | --- | --- | --- | --- | --- |
| *Searsia natalensis* (Bernh. ex Krauss) F.A.Barkley; Synonym: *Rhus natalensis* Bernh. ex C.Krauss | Anacardiaceae | Stem bark | Uganda: Fm [5] | 1 | HIV/AIDS |
| *Searsia pyroides* (Burch.) Moffett; Synonym*: Rhus vulgaris* Meikle | Anacardiaceae | Leaves | Uganda: Fm [5] | 1 | HIV/AIDS |
| *Securidaca longepedunculata*  Fresen. | Polygalaceae | Stem bark; root; root bark; leaves; whole plant | Zambia: Fi_1_ [2]; Ethiopia: Fc [7]; Nigeria: UMi [16]; [20]; Fu [10]; Uganda: Fm [5]; Namibia: Fi_2_, Fi_3_ [19] | Fi_1_: 20; Fc:  2; UMi:  37.5; Fm:  2; Fu: 4;  Fi_2_: 32; Fi_3_:  18 | Gonorrhoea (Fi_1_, Fi_2_, Fc, Fu); Syphilis (Fi_1_, Fi_3_, UMi); HIV/AIDS (Fm);  Trichomoniasis (UMi) |
| *Senegalia ataxacantha* (DC.) Kyal. & Boatwr.; Synonym: *Acacia ataxacantha* DC. | Fabaceae | Root | Zambia: Fi [3] | 50 | Gonorrhoea; Syphilis |
| *Senegalia mellifera* (Benth.) Seigler & Ebinger; Synonym: *Acacia mellifera* (M.Vahl) Benth. | Fabaceae | Stem bark and root | Zambia: Fi [2] | 35 | Syphilis |
| *Senegalia polyacantha* (Willd.) Seigler & Ebinger; Synonym: *Acacia polyacantha* Willd. | Fabaceae | Leaves and stem bark | Zambia: Fi [3] | 50 | Gonorrhoea |
| *Senegalia polyacantha* subsp. *campylacantha* (Hochst. ex A.Rich.) Kyal. & Boatwr; Synonym: *Acacia campylacantha* Hochst. ex  A.Rich. | Fabaceae | Stem bark | Uganda: Fm [5] | 1 | HIV/AIDS |

| **Botanical names** | **Families** | **Plant part used** | **Country of study: selection criteria (SC) and ethnobotanical studies (references)** | **SC index** | **Venereal diseases treated** |
| --- | --- | --- | --- | --- | --- |
| *Senegalia schweinfurthii* (Brenan & Exell) Seigler & Ebinger; Synonym: *Acacia schweinfurthii* Brenan & Exel | Fabaceae | Root, stem bark and leaves | Zambia: Fi [3] | 33.3 | Gonorrhoea; Syphilis |
| *Senna alata* (L.) Roxb.; Synonym: *Cassia alata* L. | Fabaceae | Leaves | Nigeria: UMi [16]; Fu [10]; Cameroon: Fi [6] | UMi: 45;  Fu: 4; Fi : 2.6 | Gonorrhoea (UMi, Fu); HIV/AIDS (Fi) |
| *Senna didymobotrya* (Fresen.) H.S.Irwin & Barneby | Fabaceae | Root | South Africa: FL [8] | FL_1_: 30;  FL_2_: 20; FL_3_, FL_4_: 10 | Gonorrhoea (FL_1_); Chlamydia (FL_2_); Syphilis (FL_3_); Genital warts (FL_4_) |
| *Senna italica* Mill. | Fabaceae | Root | South Africa: Fi [14] | 6.7 | Gonorrhoea |
| *Senna italica* subsp. arachoides (Burch.) Lock | Fabaceae | Root | South Africa: Ci [12] | 6 | Gonorrhoea |
| *Senna occidentalis* (L.) Link; Synonym: *Cassia occidentalis* L. | Fabaceae | Root; leaves; whole root; stem bark | Zambia: Fi [2]; RFC [4]; Ethiopia: Fc [7]; Uganda: Fm [5] | Fi: 60; Fc:  3; Fm: 1;  RFC: 0.6 | Gonorrhoea (Fi, Fc, RFC); HIV/AIDS (Fm) |
| *Senna singueana* (Delile) Lock; Synonym: *Cassia singueana* Delile (Lock) | Fabaceae | Leaves | Tanzania: Ci [1] | 13 | Gonorrhoea |
| *Senna* spp. | Fabaceae | Whole plant | Nigeria: Fi [17] | 1 | Gonorrhoea |
| *Sesbania sesban* (L.) Merr. | Fabaceae | Leaves; stem wood | Uganda: Fm [5] | 2 | HIV/AIDS |
| *Shirakiopsis elliptica* (Hochst.)  Esser; Synonym: *Sapium ellipticum* Pax | Euphorbiaceae | Root bark | Uganda: Fm [5] | 1 | HIV/AIDS |

| **Botanical names** | **Families** | **Plant part used** | **Country of study: selection criteria (SC) and ethnobotanical studies (references)** | **SC index** | **Venereal diseases treated** |
| --- | --- | --- | --- | --- | --- |
| *Sida alba* L. | Malvaceae | Root and leaves | Zambia: Fi [3] | 20 | Gonorrhoea |
| *Sida cuneifolia* Roxb. | Malvaceae | Leaves | Uganda: Fm [5] | 1 | HIV/AIDS |
| *Sigesbeckia orientalis* L. | Asteraceae | Leaves | Uganda: Fm [5] | 1 | HIV/AIDS |
| *Solanecio gigas* (Vatke) C.Jeffrey | Asteraceae | Root | Ethiopia: Fc [7] | 1 | Gonorrhoea |
| *Solanecio mannii* (Hook. f) C.Jeffrey | Asteraceae | Root wood | Uganda: Fm [5] | 1 | HIV/AIDS |
| *Solanum aculeatissimum* Jacq | Solanaceae | Root | South Africa: UV [18] | 0.08 | Gonorrhoea |
| *Solanum americanum* Miller | Solanaceae | Leaves and root | Ethiopia: Fc [7] | 2 | Gonorrhoea |
| *Solanum anguivi* Lam. | Solanaceae | Root | Ethiopia: Fc [7] | 2 | Gonorrhoea |
| *Solanum campylacanthum* | Solanaceae | Fruit; root | South Africa: Ci [12]; Fi_2_ | Ci: 6; Fi_1_: | Gonorrhoea (Ci, Fi_2_); |
| Hochst. ex A.Rich.; Synonym: |  |  | [14]; Zambia: Fi_1_ | 24; Fi_2_: 6.7; | HIV/AIDS (Fi_1,_ RFC) |
| *Solanum panduriforme* Drège |  |  | [2]; RFC (Nyirenda and | RFC: 0.2. |  |
| ex Dunal |  |  | Chipuwa, 2024) |  |  |
| *Solanum dasyphyllum*  Schumach. & Thonn. | Solanaceae | Root | Ethiopia: Fc [7] | 1 | Gonorrhoea |
| *Solanum elaeagnifolium* Cav. | Solanaceae | Root; stem gall | South Africa: FL [8] | FL_1_: 37.5; | Gonorrhoea (FL_1_); |
|  |  |  |  | FL_2_, FL_3_: | Chlamydia (FL_2_); |
|  |  |  |  | 18.8; FL_4_: | Syphilis (FL_3_); Genital |
|  |  |  |  | 12.5; FL_5_: 6.3 | warts (FL_4_); HIV/AIDS |
|  |  |  |  |  | (FL_5_) |
| *Solanum incanum* L. | Solanaceae | Root; root bark; flower | Ethiopia: Fc [7] | Fm: 1; Fc:  4 | Gonorrhoea (Fc); HIV/AIDS (Fm) |

| **Botanical names** | **Families** | **Plant part used** | **Country of study: selection criteria (SC) and ethnobotanical studies (references)** | **SC index** | **Venereal diseases treated** |
| --- | --- | --- | --- | --- | --- |
| *Solanum lycopersicum* L.; Synonym: *Lycopersicon esculentum* Mill. | Solanaceae | Leaves | Ethiopia: Fc [7] | 1 | Gonorrhoea |
| *Solanum mauritianum* Scop. | Solanaceae | Root | South Africa: FL [8] | FL_1_: 36.4;  FL_2_: 27.3;  FL_3_: 18.2;  FL_4_: 9.1 | Gonorrhoea (FL_1_); Syphilis (FL_2_); Chlamydia (FL_3_); HIV/AIDS (FL_4_) |
| *Sonchus oleraceus* L. | Asteraceae | Root | Kenya: ICf [15] | 0.4 | Gonorrhoea |
| *Sorghum bicolor* (L.) Moench | Poaceae | Seed | Nigeria: Fu [10] | 1 | Gonorrhoea; Syphilis |
| *Spathodea campanulata*  P.Beauv. | Bignoniaceae | Stem bark | Uganda: Fm [5] | 2 | HIV/AIDS |
| *Spondias mombin* L. | Anacardiaceae | Stem bark; leaves | Nigeria: Fc [11]; UMi [16] | UMi: 26.3;  Fc: 46 | Gonorrhoea (Fc); Syphilis (UMi) |
| *Sporobolus pyramidalis*  P.Beauv. | Poaceae | Root | Kenya: ICf [15] | 0.4 | Gonorrhoea |
| *Steganotaenia araliacea*  Hochst. | Apiaceae | Whole root; stem bark | Uganda: Fm [5] | 3 | HIV/AIDS |
| *Stephania abyssinica* Walp. | Menispermaceae | Root | Ethiopia: Fc [7] | 1 | Gonorrhoea |
| *Strophanthus eminii* Asch. ex Pax | Apocynaceae | Stem bark | Tanzania: Ci [1] | 21.7 | Syphilis |
| *Strophanthus sarmentosus* DC. | Apocynaceae | Bark | Nigeria: Fc [11] | 31 | Gonorrhoea |
| *Strychnos cocculoides* Baker | Loganiaceae | Root | Zambia: Fi [2]; RFC [4] | Fi: 75;  RFC: 0.4 | Gonorrhoea (Fi, RFC) |
| *Strychnos innocua* Delile | Loganiaceae | Root fruit pulp | Zambia: Fi_1_ [2]; Fi_2_ [3] | Fi_1_: 50; Fi_2_:  20 | Gonorrhoea (Fi_1_, Fi_2_); Chlamydia (Fi_1_) |

| **Botanical names** | **Families** | **Plant part used** | **Country of study: selection criteria (SC) and ethnobotanical studies (references)** | **SC index** | **Venereal diseases treated** |
| --- | --- | --- | --- | --- | --- |
| *Strychnos potatorum* L.f. | Loganiaceae | Root; leaves | Zambia: Fi_1_ [2]; Fi_2_ [3] | Fi_1_: 30; Fi_2_:  40 | Syphilis |
| *Suregada procera* (Prain) Croizat | Euphorbiaceae | Root | Ethiopia: Fc [7] | 1 | Gonorrhoea |
| *Synsepalum dulcificum*  (Schumach. & Thonn.) Daniell | Sapotaceae | Root | Nigeria: Fc [11] | 45 | Gonorrhoea |
| *Syzygium guineense* DC. | Myrtaceae | Root, stem and leaves | Ethiopia: Fc [7] | 1 | Gonorrhoea |
| *Terminalia avicennioides* Guill. & Perr. | Combretaceae | Stem bark | Nigeria: UMi [16] | 25 | Gonorrhoea |
| *Terminalia hylodendron* (Mildbr.) Gere & Boatwr.; Synonym: *Pteleopsis hylodendron* Mildbr. | Combretaceae | Stem bark | Cameroon: Fi [6] | 6 | HIV/AIDS |
| *Terminalia kaiseriana*  F.Hoffm. | Combretaceae | Root | Tanzania: Ci [1] | 30.4 | Gonorrhoea; Syphilis |
| *Terminalia prunioides*  M.A.Lawson | Combretaceae | Root bark | Zambia: Fi [3] | 83.3 | Gonorrhoeae; Syphilis; HIV/AIDS |
| *Terminalia schimperiana* Hochst. ex Engl. & Diels; Synonym: *Terminalia glaucescens* Planch. ex Benth. | Combretaceae | Root bark | Uganda: Fm [5] | 1 | HIV/AIDS |
| *Terminalia sericea* Burch. ex DC. | Combretaceae | Stem bark; root and leaves | Zambia: Fi [2]; Tanzania: Ci [1] | Fi: 80; Ci:  13 | Gonorrhoea (Ci, Fi); Syphilis (Fi) |
| *Terminalia superba* Engl. & Diels | Combretaceae | Stem bark | Cameroon: Fi [6] | 2.6 | HIV/AIDS |

| **Botanical names** | **Families** | **Plant part used** | **Country of study: selection criteria (SC) and ethnobotanical studies (references)** | **SC index** | **Venereal diseases treated** |
| --- | --- | --- | --- | --- | --- |
| *Tetradenia urticifolia* (Baker) Phillipson | Lamiaceae | Leaves | Uganda: Fm [5] | 1 | HIV/AIDS |
| *Tetrapleura tetraptera*  (Schumach. & Thonn.) Taub. | Fabaceae | Seed | Nigeria: UMi [16] | 5 | Gonorrhoea |
| *Theobroma cacao* L. | Malvaceae | Bark | Nigeria: Fu [10] | 2 | Gonorrhoea |
| *Tribulus terrestris* L. | Zygophyllaceae | Whole plant | South Africa: Ci [12] | 3 | HIV/AIDS |
| *Trichilia dregeana* Harv. & Sond. | Meliaceae | Root | Ethiopia: Fc [7] | 1 | Gonorrhoea |
| *Trichilia emetic* Vahl | Meliaceae | Leaves and stem bark; root | Zambia: Fi [2]; RFC [4] | Fi: 20;  RFC: 0.2 | Gonorrhoea; Syphilis (Fi, RFC) |
| *Triumfetta* spp. | Malvaceae | Root | South Africa: Ci [12] | 3 | Chlamydia |
| *Tropaeolum majus* L. | Tropaeolaceae | Leaves | Uganda: Fm [5] | 1 | HIV/AIDS |
| *Urtica dioica* L. | Urticaceae | Whole plant | Ethiopia: Fc [7] | 1 | Gonorrhoea |
| *Urtica simensis* Hochst. ex A.Rich. | Urticaceae | Leaves and root | Ethiopia: Fc [7] | 3 | Gonorrhoea |
| *Uvaria afzelii* Scott Elliot | Annonaceae | Root | Nigeria: UMi [16] | 25 | Gonorrhoea |
| *Vachellia gerrardii* (Benth.) P.J.H.Hurter; Synonym: *Acacia gerrardii* Benth. | Fabaceae | Stem bark | Uganda: Fm [5] | 2 | HIV/AIDS |
| *Vachellia nilotica* (L.) P.J.H.Hurter & Mabb.; Synonym: *Acacia nilotica* (L.) Willd. ex Delile | Fabaceae | Leaves; twig; root and stem bark | Zambia: Fi_1_ [2]; Fi_2_ [3] | Fi_1_: 25; Fi_2_:  66.7 | Gonorrhoea (Fi_1_); Chlamydia (Fi_2_) |

| **Botanical names** | **Families** | **Plant part used** | **Country of study: selection criteria (SC) and ethnobotanical studies (references)** | **SC index** | **Venereal diseases treated** |
| --- | --- | --- | --- | --- | --- |
| *Vachellia seyal* (Delile) P.J.H.Hurter; Synonym: *Acacia seyal* Delile | Fabaceae | Stem bark | Uganda: Fm [5] | 2 | HIV/AIDS |
| *Vachellia sieberiana* (DC.) Kyal. & Boatwr.; Synonym: *Acacia sieberiana* DC. | Fabaceae | Stem bark | Uganda: Fm [5] | 2 | HIV/AIDS |
| *Verbena officinalis* L. | Verbenaceae | Root | Ethiopia: Fc [7] | 1 | Gonorrhoea |
| *Vernonia adoensis* Walp. | Asteraceae | Leaves | Uganda: Fm [5] | 1 | HIV/AIDS |
| *Vernonia amygdalina* Delile | Asteraceae | Leaves | Nigeria: Fi [17] | 1 | Gonorrhoea |
| *Vigna vexillata* var. *vexillata*; Synonym: *Vigna dinteri* Harms | Fabaceae | Root | Namibia: Fi [19] | 7 | Genital herpes |
| *Vitex ferruginea* Schumach. & Thonn. | Lamiaceae | Root bark | Uganda: Fm [5] | 1 | HIV/AIDS |
| *Vitellaria paradoxa* C.F.Gaertn. | Sapotaceae | Bark | Nigeria: Fi [17] | 1 | Gonorrhoea |
| *Vitex petersiana* Klotzsch | Lamiaceae | Leaves | Zambia: Fi [3] | 23.3 | Gonorrhoea |
| *Waltheria indica* L. | Sterculiaceae | Stem | Namibia: Fi [19] | 42 | Syphilis |
| *Warburgia salutaris* (G.Bertol.) Chiov. | Canellaceae | Stem bark; root bark | Uganda: Fm [5] | 4 | HIV/AIDS |
| *Withania somnifera* (L) Dunal | Solanaceae | Root | South Africa: UV [18] | 0.08 | Gonorrhoea |
| *Ximenia americana* L. | Olacaceae | Stem; stem bark; root; leaves and fruit | Zambia: Fi_1_ [2]; Fi_2_ [3]; RFC [4]; Namibia: Fi_3_ [19]; Tanzania: Ci [1] | Fi_1_: 25; Fi_2_:  83.3; Fi_3_:  32; Ci: 13;  RFC: 0.2 | Gonorrhoea (Fi_1_, Fi_2_, Fi_3_, Ci, RFC); Syphilis (Fi_1_) |

| **Botanical names** | **Families** | **Plant part used** | **Country of study: selection criteria (SC) and ethnobotanical studies (references)** | **SC index** | **Venereal diseases treated** |
| --- | --- | --- | --- | --- | --- |
| *Ximenia caffra* Sond. | Olacaceae | Root and leaves; stem | Tanzania: Ci [1]; Namibia: Fi [19] | Ci: 39.1;  Fi: 67 | Gonorrhoea (Ci, Fi); Syphilis (Fi) |
| *Xylopia aethiopica* (Dunal) A.Rich. | Anonnaceae | Stem bark; leaves | Nigeria: Fi [20]; UMi [16] | UMi: 18.8;  Fi: N/A | Gonorrhoea (UMi); HIV/AIDS (Fi) |
| *Zanthoxylum capense* Harv. | Rutaceae | Root | South Africa: Ci [12] | 3 | HIV/AIDS |
| *Zanthoxylum chalybeum* Engl. | Rutaceae | Stem bark; stem wood; whole root; root bark; root wood | Uganda: Fm [5]; Tanzania: Ci [1] | Fm: 1; Ci:  21.7 | Gonorrhoea (Fm); HIV/AIDS (Ci) |
| *Zanthoxylum gilletii* (De Wild.) P.G.Waterman; Synonym: *Fagara macrophylla* Engl. | Rutaceae | Bark | Nigeria: Fc ,; Uganda: Fm [5] | Fc: 31; Fm:  1 | Gonorrhoea (Fc); HIV/AIDS (Fm) |
| *Zanthoxylum humile*  (E.A.Bruce) P.G.Waterman | Rutaceae | Root | South Africa: Ci [12] | 6 | HIV/AIDS |
| *Zanthoxylum leprieurii* Guill. & Perr. | Rutaceae | Stem bark; stem wood; whole root; root bark; root wood | Uganda: Fm [5] | 1 | HIV/AIDS |
| *Zea mays* L. | Poaceae | Grains | Cameroon: Fi [6] | 2 | HIV/AIDS |
| *Zehneria scabra* Sond. | Cucurbitaceae | Leaves | Cameroon: Fi [6]; Uganda: Fm [5] | Fi: 4.6; Fm:  2 | HIV/AIDS |
| *Zingiber officinale* Roscoe | Zingiberaceae | Rhizome | Cameroon: Fi [6] | 2 | HIV/AIDS |
| *Ziziphus mauritiana* Lam. | Rhamnaceae | Fruit | Zambia: Fi [3] | 40 | Gonorrhoea; Syphilis |
| *Ziziphus mucronata* Willd. | Rhamnaceae | Root; stem bark; leaves | South Africa: Fi_1_ [14]; Ci_2_ [12]; Tanzania: Ci_1_ [1]; Namibia: Fi_4_ [19]; Zambia: Fi_2_[2]; Fi_3_ [3]; Botswana: Fi_5_ [13] | C_1_: 13; C_2_:  18; Fi_1_: 6.7;  Fi_2_: 40; Fi_3_:  83.3; Fi_4_: 71; Fi_5_:  N/A | Gonorrhoea (Ci_2_, Fi_1_, Fi_3_, Fi_4_); Chlamydia (Ci_1_, Ci_2_, Fi_2_); Genital herpes (Fi_5_) |

**Key:** Ci: citation index **|** Fc: frequency of citation **|** Fi: frequency index **|** FL: fidelity level **|** Fm: frequency of mention **|** Fu: frequency of use **|** ICf: informant consensus factor **|** UMi: use mention index. **|** *Bidens* sp.*: not *Bidens pilosa* L. **|** *Mentha* sp.*: close to *Mentha piperata* L. (*Mentha × piperata*) **|** **Protea caffra subsp. caffra;* Synonym: *Protea caffra* Meisn. and **Ipomoea hildebrandtii* Vatke could only be verified with the Medicinal Plant Names Services portal **|** Ci (%) = Fi (%) = UMi (%) = $\frac{Na}{Nb}\times100$ (expressed as a quantitative indicator to determine the importance of an ethnomedicine within a community, where Na is the number of informants or traditional health practitioners that cited a plant as an ethnomedicine, while Nb is the total number of intervewed informants or traditional health practitioners). **|** FL (%) = $\frac{Np}{N}\times100$ (expressed as a quantitative indicator to determine the importance of an ethnomedicine for a particular use within a community, where Np is the number of informants or traditional health practitioners that cited a plant as an ethnomedicine for a particular venereal disease, while N is the total number of use-report of ethnomedicine for any purpose). **|** Fc = Fm = Fu (expressed as the number of times the use of an ethnomedicine is cited or mentioned for a particular venereal disease). **|** ICf = $\frac{(Nur-Nt)}{(Nur-1)}$ (expressed as quantitative indicator to determine the level of agreement among informants about the use of plant for a particular venereal disease, where Nur is the number of use reports from informants or traditional health practitioners for a specific venereal disease, while Nt represents the number of varieties species or subspecies used for that venereal disease). | N/A, i.e. not applicable - refers to the ethnobotanical/citation index not accounted for in the source paper.

**References**

1. Kacholi DS, Mvungi AH: Ethnobotanical survey of medicinal plants used by traditional healers in managing gonorrhoea and syphilis in Urambo District, Tabora Region, Tanzania. *Journal of Herbs, Spices & Medicinal Plants* 2022, 28:179-192.

2. Chinsembu KC: Ethnobotanical study of medicinal flora utilized by traditional healers in the management of sexually transmitted infections in Sesheke District, Western Province, Zambia. *Revista Brasileira de Farmacognosia* 2016, 26:268-274.

3. Chinsembu KC: Plants used to manage HIV/AIDS-related diseases in Livingstone, Southern Province, Zambia. *Evidence-Based Complementary and Alternative Medicine* 2016:14.

4. Nyirenda J, Chipuwa M: Ethnobotanical study of herbs and medicinal plants in Zambia. *Phytomedicine Plus* 2024, 4.

5. Lamorde M, Tabuti JRS, Obua C, Kukunda-Byobona C, Lanyero H, Byakika-Kibwika P, Bbosa GS, Lubega A, Ogwal-Okeng J, Ryan M *et al*: Medicinal plants used by traditional medicine practitioners for the treatment of HIV/AIDS and related conditions in Uganda. *Journal of Ethnopharmacology* 2010, 130:43-53.

6. Noumi E, Manga P: Traditional medicines for HIV/AIDS and opportunistic infections in North-West Cameroon: Case of skin infections. *American Journal of Tropical Medicine & Public Health* 2011, 1:44-64.

7. Bizuayehu B, Garedew B: A review on the ethnobotanical study of medicinal plants used for the treatment of gonorrhea disease in Ethiopia. *Indian Journal of Natural Products and Resources* 2018, 9:183-193.

8. Maema LP, Potgieter M, Samie A: Ethnobotanical survey of invasive alien plant species in the treatment of sexually transmitted infections in Waterberg District, South Africa. *South Africa Journal of Botany* 2019, 19:391-400.

9. Mbambala SG, Tshisikhawe M, Masevhe NA: Invasive alien plants used in the treatment of HIV/AIDS-related symptoms by traditional healers of Vhembe Municipality, Limpopo Province, South Africa. *African Journal of Traditional, Complementary and Alternative Medicines* 2017, 14:11.

10. Gbadamosi I, Egunyomi A: Ethnobotanical survey of plants used for the treatment and management of sexually transmitted infections in Ibadan, Nigeria. *Ethnobotany Research and Applications* 2014, 12:659-669.

11. Ajibesin K, Umoh UF, Bala DN: The use of medicinal plants to treat sexually transmitted diseases in Nigeria: Ethnomedicinal survey of Niger Delta Region. *International Journal of Green Pharmacy* 2011, 5:181.

12. Semenya SS, Potgieter MJ, Erasmus LJC: Bapedi phytomedicine and their use in the treatment of sexually transmitted infections in Limpopo Province, South Africa. *African Journal of Pharmacy and Pharmacology* 2013, 7:250-262.

13. Richard K, Andrae-Marobela K, Tietjen I: An ethnopharmacological survey of medicinal plants traditionally used by the BaKalanga people of the Tutume subdistrict in Central Botswana to manage HIV/AIDS, HIV-associated conditions, and other health conditions. *Journal of Ethnopharmacology* 2023, 316.

14. Erasmus LJC, Potgieter MJ, Semenya S, Lennox S: Phytomedicine versus gonorrhoea: the Bapedi experience. *African Journal of Traditional, Complementary and Alternative Medicines* 2012, 9:17.

15. Njoroge G, Bussmann R: Ethnotherapeutic management of sexually transmitted diseases (STDs) and reproductive health conditions in Central Province of Kenya. *Indian Journal of Traditional Knowledge* 2009, 8:255-261.

16. Omilani A: Ethnobotanical survey of the medicinal plants used in the treatment of sexually transmitted diseases in Ibadan, Oyo State, Nigeria. *ScienceOpen* 2021.

17. Salami KD, Alao JS, Abubakar U, Adam F: Ethno-botanical survey of plants applied for the treatment of sexually transmitted diseases in the Sudan Savannah Region of Nigeria. *Journal of Advances in Biology & Biotechnology* 2022, 25:55-63.

18. Ngobeni B, Manduna IT, Malebo NJ, Mashele SS: Phytotherapy for sexually transmitted infections in Thaba ‘Nchu, Free State Province, South Africa. *Pharmacognosy Journal* 2023, 15:21-30.

19. Hedimbi M, Chinsembu KC: Ethnomedicinal study of plants used to manage HIV/AIDS-related disease conditions in the Ohangwena Region, Namibia. *International Journal of Medicinal Plant Research* 2012, 1:4-11.

20. Ohemu T, Agunu AA, Olotu PN, Ajima U, Dafam D, Azila JJ: Ethnobotanical survey of medicinal plants used in the traditional treatment of viral infection in Jos, Plateau State, Nigeria. *International Journal of Medicinal and Aromatic Plants* 2014, 4:74-81.
